# Fault Monitoring Based on the VLSW-MADF Test and DLPPCA for Multimodal Processes

**DOI:** 10.3390/s23020987

**Published:** 2023-01-14

**Authors:** Shu Wang, Yicheng Wang, Jiarong Tong, Yuqing Chang

**Affiliations:** College of Information Science and Engineering, Northeastern University, Shenyang 110819, China

**Keywords:** multimode process, mode identification, process monitoring, statistical modeling

## Abstract

Actual industrial processes often exhibit multimodal characteristics, and their data exhibit complex features, such as being dynamic, nonlinear, multimodal, and strongly coupled. Although many modeling approaches for process fault monitoring have been proposed in academia, due to the complexity of industrial data, challenges remain. Based on the concept of multimodal modeling, this paper proposes a multimodal process monitoring method based on the variable-length sliding window-mean augmented Dickey–Fuller (VLSW-MADF) test and dynamic locality-preserving principal component analysis (DLPPCA). In the offline stage, considering the fluctuation characteristics of data, the trend variables of data are extracted and input into VLSW-MADF for modal identification, and different modalities are modeled separately using DLPPCA. In the online monitoring phase, the previous moment’s historical modal information is fully utilized, and modal identification is performed only when necessary to reduce computational cost. Finally, the proposed method is validated to be accurate and effective for modal identification, modeling, and online monitoring of multimodal processes in TE simulation and actual plant data. The proposed method improves the fault detection rate of multimodal process fault monitoring by about 14% compared to the classical DPCA method.

## 1. Introduction

The requirements for safety in industrial production have increased in rigor with the rapid expansion of modern industry. Accurate fault monitoring, diagnosis, and treatment of industrial processes contribute to the normal operation of industrial production and the prevention of further accident spreading. However, the modern industrial structure is complex, with numerous subsystems and variables that are frequently dynamic, non-linear, and highly correlated. Furthermore, due to changes in actual production requirements, industrial processes frequently contain multiple stable and transitional modes. These characteristics of complex industrial processes make monitoring and diagnosing faults more difficult.

In recent decades, data-driven methods [1] have been widely used in the field of industrial technology. This is because a data-driven method does not need to establish an accurate mathematical model or possess too much prior knowledge. Such methods mainly depend on a large amount of process data to analyze and monitor [2,3,4] the operation of the system or equipment under study. Currently, the mainstream research methods of fault monitoring and diagnosis technology primarily include data-driven multivariate statistics and novel machine learning or artificial intelligence methods. Aldrich and Auret provided a comprehensive review of unsupervised machine learning-based process monitoring methods [5]. Fan trained the autoencoder using offline normal data by building the structure of a neural network and then used it for online fault detection [6].

Classical data-driven approaches include PCA [7], ICA [8], PLS [9], and other methods. Each of these methods has its own strengths and drawbacks. Traditional PCA is more suitable for production process data that are linear and satisfy Gaussian distribution, while it performs poorly for data that are strongly nonlinear, dynamic, and coupled. Therefore, many scholars have made improvements based on PCA and proposed many methods. For example, Lee proposed KPCA to handle nonlinear data for industrial process data presenting strong nonlinearity [10]. Ku considered the serial correlation of process data and proposed DPCA, which performs better for highly dynamic data [11]. Bakshi proposed MSPCA to solve the multiscale problem [12]. Harrou combined the multivariate exponentially weighted moving average (MEWMA) monitoring scheme with PCA modeling to improve anomaly detection performance [13], and so on.

Considering the importance of local data features represented by data neighborhood information, manifold learning is proposed to provide a novel perspective to preserve the local features of data [14]. According to this method, data are formed by mapping a low-dimensional manifold onto a high-dimensional space. As a result, the low-dimensional data can uniquely represent the original data. Extraction of low-dimensional manifolds from high-dimensional data is accomplished by first establishing a local reduced-dimensional mapping relationship and then attempting to generalize the local mapping relationship to the global. Isomap [15], Laplacian eigenmaps (LE) [16], and locality-preserving projections (LPP) [17] are popular manifold learning techniques. Currently, manifold learning has been applied to process monitoring in industrial processes. By using LPP in combination with PCA, Yu proposed a principal component analysis method (LGPCA) for local and global applications [18]. Luo further revealed the relationship between LPP and PCA and proposed a novel dimensionality reduction algorithm, GLPP, which aims to preserve the global and local structure of the dataset by solving a biobjective optimization function [19]. Wu used PCA, LPP, and isometric feature mapping (ISOMAP) to fuse features extracted from vibration signals for fault diagnosis [20]. These methods have proven to outperform PCA-based and LPP-based monitoring methods.

However, actual industrial process variables are highly dynamic and have characteristics such as autocorrelation and intercorrelation. Traditional methods are difficult to effectively model highly dynamic data, which may result in false alarms in online monitoring. Moreover, due to the changing input point of the working condition and changes in the underlying raw material, the operating state of the industrial process will change to varying degrees, thus showing several different modes. Most data-driven methods operate in a single stable mode but perform poorly on multimodal process data. To accurately model and monitor multimodal processes, some methods have been proposed and practiced in the past. There are two main ideas: 1—Overall modeling [21,22,23] entails using the same model to describe different modes. 2—Multimodal modeling [24,25,26] involves describing the process characteristics of each mode by building local models for different stable modes. The goal of overall modeling is to build models that describe the different structures of all modes, such as global PCA models. However, this kind of method can lead to the deterioration of monitoring accuracy for some modes. False alarms may even occur. Multimodal modeling-based approaches model different modes separately. Modeling individual modes is more accurate than overall modeling [27,28].

The main contributions of this paper are as follows: first, an offline mode identification method based on the variable-length sliding window-mean augmented Dickey–Fuller (VLSW-MADF) test is proposed. The commonly used offline mode identification work is based on the trend of variation of each variable for mode classification. The method innovatively uses the smoothness of the data as the basis for stable and transitional mode identification. Compared with other multimodal classification methods, the proposed method in this paper is more intuitive, and the starting position of transition modes can be determined more accurately. Secondly, this paper improves the traditional data-driven fault monitoring method and proposes a novel fault monitoring method DLPPCA. Many scholars have studied the modeling of transition modes [29]; however, these methods often do not focus on the transition modes themselves. DLPPCA performs well on dynamic transition mode data, can accurately model and monitor both transition and stable modes, and is more suitable for modeling and monitoring multimodal processes. Finally, this paper proposes a novel and less computationally intensive online modal identification method. The traditional online modal identification method requires traversing all offline models [30]. When the number of modalities in the process is large, the computational effort is too large. The modal identification method proposed in this paper is based on matching value calculation and uses an offline matching matrix with the same sample length as the online data for modal identification, which reduces the computational effort and improves the accuracy at the same time.

The paper is organized as follows: Section 2 describes process monitoring based on DLPPCA. Section 3 describes the offline mode recognition and modeling steps based on the VLSW-MADF test and DLPPCA. Section 4 describes the online mode recognition and monitoring strategies proposed in this paper. Section 5 uses the TE process and actual power plant data to simulate and verify the validity and correctness of the methods presented in this paper. Finally, the conclusion of this article is presented in Section 6. To avoid confusion among the many symbols, we have listed a nomenclature.

## 2. Process Monitoring Based on DLPPCA

PCA is a widely used data-driven method that performs well on data feature extraction tasks and is often applied for process monitoring in industrial practice [31,32,33,34,35]. However, this method often ignores the local structure underlying data, resulting in the loss of potential information from such structures. Locality-preserving projection (LPP) is a manifold learning method that maintains the local structure of data and can restore a low-dimensional manifold structure from high-dimensional sample data [36,37,38]. At present, scholars use LPP in combination with PCA [39,40], but the statistical model established by this combination method is static; that is, it assumes that the current process is time-invariant. In real industrial processes, process variables have dynamic characteristics of autocorrelation and cross-correlation. Since static PCA is unable to extract dynamic relationships from the data, autocorrelation and cross-correlation are mixed together, which makes it difficult for traditional PCA to reveal what type of relations among the measured variables. Direct application of traditional fault monitoring methods to dynamic data may lead to misleading results (real-time statistics exceeding real-time thresholds, resulting in false fault alarms). Therefore, we must consider the process data serial correlations to implement an efficient monitoring method.

Therefore, a process monitoring method based on dynamic locality-preserving principal component analysis (DLPPCA) is presented to solve the above problems. DLPPCA first constructs an extended matrix to associate adjacent sample points, and this solves the problem of strong correlation among the sample points in a dynamic process. LPP and PCA are combined to extract the maximum variance information of the manifold structure. This algorithm not only solves the problem of traditional data-driven methods having difficulty modeling due to the strong dynamic natures of industrial processes, but also makes up for the disadvantage of PCA or LPP being used alone by combining LPP with PCA. The steps for DLPPCA are as follows:

First, we assume that the sample set is $X\in {\mathbb{R}}^{n\times m}$ (m is the number of variables, and n is the number of samples) and that the sample set X has been standardized.

(1) $$X={\left[x_{m}\left(1\right),x_{m}\left(2\right),\ldots ,x_{m}\left(n\right)\right]}^{T}\in {\mathbb{R}}^{n\times m}$$

The original sample set X is dynamically expanded into a new matrix $X^{*}$ by adding the time lag values of the variables using the “time lag shift” method proposed by Ku [11]. The sample set: $X\in {\mathbb{R}}^{n\times m}$ is expanded to:

(2) $$X^{*}=\left[X\left(t\right),X\left(t-1\right),\cdots X\left(t-l\right)\right]\in {\mathbb{R}}^{\left(n-l\right)\times m\left(l+1\right)}$$

where l is the number of lags. It is selected by experience and should not be too large; generally, l=1,2. Where $X\left(t\right)$ is the first column of the dynamic expansion matrix and $x^{T}\left(t\right)$ is the m-dimensional observation vector in the sample set at moment t.

(3) $$X\left(t\right)={\left[x^{T}\left(t\right),x^{T}\left(t-1\right),\cdots ,x^{T}\left(t-n+l\right)\right]}^{T}$$

Next, the low-dimensional manifold structure of the data is extracted. The goal of manifold feature extraction is to find a projection matrix $\;A=\left[\alpha_{1},\alpha_{2},\ldots ,\alpha_{k}\right]$ $k<m\left(l+1\right)\;$ such that the extracted low-dimensional manifold $F=\left[f_{1},f_{2},\ldots ,f_{n}\right]\in {\mathbb{R}}^{k\times \left(n-l\right)}$ retains a local structure similar to that of $X^{*}$. Then, we have the following objective function:

(4) $$X\frac{1}{2}min\overset{n}{\underset{i,j}{\sum }}{\left(f_{i}-f_{j}\right)}^{2}W_{ij}\;=\frac{1}{2}min\overset{n}{\underset{i,j}{\sum }}{\left(A^{T}X\left(i\right)-A^{T}X\left(j\right)\right)}^{2}W_{ij}\;=min\overset{n}{\underset{i}{\sum }}A^{T}X\left(i\right)D_{ii}X{\left(i\right)}^{T}A-min\overset{n}{\underset{i,j}{\sum }}A^{T}X\left(i\right)W_{ij}X{\left(j\right)}^{T}A\;=minA^{T}X^{*}\left(D-W\right)X^{*}{}^{T}A$$

where W is a $W_{ij}$ relational matrix

(5) $$W_{ij}=\left\{\begin{array}{c} e^{-\frac{∥X\left(i\right)-X\left(j\right){∥}^{2}}{t}}\;\;\;\;\;\;\;\;\;\;X\left(i\right)\in N_{k}\left(X\left(j\right)\right)\;or\;X\left(j\right)\in N_{k}\left(X\left(i\right)\right)\\ 0\;\;\;otherwise \end{array}\right.$$

and D is an n×n diagonal matrix. The diagonal elements are $D_{ii}=\overset{n}{\underset{j=1}{\sum }}W_{ij}$ and can be used to indicate the importance of each sample. To ensure that the objective function is solvable, a restriction $FDF^{T}=I$ or $A^{T}X^{*}DX^{*}{}^{T}A=I$ needs to be added. We define a Laplacian matrix $L_{P}$: $L_{P}=D-W$. Equation (4) is converted to an optimization problem, as shown below:

(6) $$minA^{T}X^{*}L_{P}X^{*}{}^{T}A\;s.t.A^{T}X^{*}DX^{*}{}^{T}A=I$$

Equation (6) is equivalent to solving the generalized eigenvalue problem shown below:

(7) $$X^{*}L_{P}X^{*}{}^{T}\alpha =\lambda X^{*}DX^{*}{}^{T}\alpha \;s.t.A^{T}X^{*}DX^{*}{}^{T}A=I$$

The projection matrix A is composed of the eigenvectors corresponding to the k minimum generalized eigenvalues obtained. Thus, the extracted low-dimensional manifold $F=A^{T}X^{*}$ is obtained.

Finally, the principal components of manifold F are extracted by PCA. The covariance matrix ∑ of the low-dimensional manifold F is as follows:

(8) $$\sum =cov\left(F\right)=\frac{F{\left(F\right)}^{T}}{n-l-1}$$

Eigenvalue decomposition is performed on the resulting covariance matrix as follows:

(9) $$\sum p_{i}=\lambda^{*}p_{i}$$

The projection matrix P is obtained by taking the eigenvectors corresponding to the d largest eigenvalues. Finally, the feature data extracted by DLPPCA are obtained:

(10) $$T=P^{T}F=P^{T}A^{T}X^{*}$$

The feature data $T\in {\mathbb{R}}^{d\times \left(n-l\right)}$ from Equation (10) are called the matching matrix. This matching matrix will be used later for online modal recognition.

The above derivation explains the basic principles of DLPPCA. Statistics and confidence limits also need to be built to monitor an industrial process. This paper is implemented with the $T^{2}$ and SPE statistics. Among them, the $T^{2}$ statistic represents the fluctuations of model variables, and the SPE statistic measures the goodness of fit of the constructed model. Once the statistics of the online data exceed the corresponding confidence limits calculated from the normal offline data, the current process is considered to have a fault situation.

The $T^{2}$ statistic is as follows:

(11) $$T^{2}=f^{T}P\Lambda^{-1}P^{T}f$$

where $f^{\left(k\times 1\right)}\in F$ and Λ is a diagonal matrix composed of the largest d in $\lambda^{*}$.

The $T^{2}$ statistic obeys the F distribution, so the confidence limit for $T^{2}$ is:

(12) $$T_{\alpha }{}^{2}=\frac{k({\left(n-l\right)}^{2}-1)}{\left(n-l\right)\left(n-k\right)}F_{\alpha }\left(k,n-l-k\right)$$

The SPE statistic is as follows:

(13) $$SPE={\left(f\right)}^{T}\left(I-PP^{T}\right)f$$

The confidence limit for the SPE is:

(14) $$SPE_{\alpha }=\theta_{1}{\left[\frac{c_{\alpha }h_{0}\sqrt{2\theta_{2}}}{\theta_{1}}+1+\frac{\theta_{2}h_{0}\left(h_{0}-1\right)}{\theta_{1}{}^{2}}\right]}^{\frac{1}{h_{0}}}$$

where

(15) $$\theta_{r}=\sum_{j=k+1}^{m}\lambda^{*}{}_{j}^{r}\left(r=1,2,3\right)$$

(16) $$h_{0}=1-\frac{2\theta_{1}\theta_{3}}{3\theta_{2}{}^{2}}\;$$

1−α represents the confidence level, which is 0.99 for this article.

## 3. Offline Mode Identification Based on the VLSW-MADF Test and Modeling

Multimodal processes contain different stationary and transition modes [41]. The stable mode mentioned in this paper refers to the industrial production process in a smooth working condition for a period of time. A stable mode means most of the time that the process data for that time series fluctuates around a stable central level. The nonstationary mode is the state of the production process when it transitions from one operating condition to another. The process data in this time series tend to have a clear upward or downward trend. Moreover, time series with nonstationary states can often be differentiated to form stationary series.

Notably, the sampling data of a multimodal process are also essentially a time series. Therefore, from the perspective of data stationarity, the stable mode can be considered as the current process data is in a stationary state, while the transition mode can be considered as the current process data is in a nonstationary state. From this point of view, this paper presents a method of pattern recognition based on the VLSW-MADF test. The method uses the stationarity of the process data as the basis for the identification of stable and transitional modes. Compared with other multimodal identification methods, the method presented in this paper starts with the intrinsic characteristics of the given data, which is more intuitive and less difficult to implement.

### 3.1. ADF Test

The augmented Dickey–Fuller (ADF) test is a stability test method that is widely used in the field of economics [42,43,44]. However, to the authors’ knowledge, the ADF test has not yet been applied in the field of process monitoring or fault diagnosis. This method makes a stationarity judgment by determining whether there is a unit root in the current data; if there is no unit root, the data are in a stationary state. If there is a unit root, the data are in a nonstationary state. The specific process of the ADF test is as follows:

Assume that we have a time series denoted by $\tilde{X}={\left[x_{1},x_{2},\ldots ,x_{n}\right]}^{T}\in {\mathbb{R}}^{n\times 1},$ (n is the number of samples). The ADF test can be completed by validating the following three models after making a first-order difference equation for $\tilde{X}$:

Model 1:

(17) $$\Delta x_{t}=\delta x_{t-1}+\overset{n}{\underset{i=1}{\sum }}\gamma_{i}\Delta x_{t-1\;}+\varepsilon_{t}$$

Model 2:

(18) $$\Delta x_{t}=\eta +\delta x_{t-1}+\overset{n}{\underset{i=1}{\sum }}\gamma_{i}\Delta x_{t-1}+\varepsilon_{t}$$

Model 3:

(19) $$\Delta x_{t}=\eta +\beta t+\delta x_{t-1}+\overset{n}{\underset{i=1}{\sum }}\gamma_{i}\Delta x_{t-1}+\varepsilon_{t}$$

where t is the time index, η is an intercept constant called a drift, β is the coefficient on a time trend, $\gamma_{i}$ is a trend term, δ is the coefficient presenting process root, and $\varepsilon_{t}$ is a white noise sequence.

The assumptions presented are as follows:

**Hypothesis 0 (H0).**  $\delta =0\left(There\;is\;a\;unit\;root,\;and\;the\;data\;are\;nonstationary\right)$.

**Hypothesis 1 (H1).**  $\delta <0\left(No\;unit\;root\;and\;the\;data\;are\;stable\right)$.

This test is performed by calculating the t-statistic for each model:

(20) $$t_{s}=\frac{\hat{\delta }-1}{{\hat{\sigma }}_{\delta }}$$

where $\hat{\delta }$ is the estimated value of δ and ${\hat{\sigma }}_{\delta }$ is the standard error.

By querying the ADF threshold table, if the obtained t-statistic is less than three confidence levels (10%, 5%, and 1%), it can be judged that the null hypothesis $H_{0}$ is rejected with 90%, 95%, and 99% confidence, respectively. If $t_{s}$ is greater than or equal to the critical value, the current data are not stationary. If t is less than or equal to the critical value, the current data are stationary.

Since it is not known in the actual test which model the data being tested conform to at this time, the ADF test first checks model 3 (Equation (19)), and then it checks model 2 (Equation (18)) and model 1 (Equation (17)) in turn. If the null hypothesis is rejected, the test stops; otherwise, the test continues. That is, when none of the three models can reject the null hypothesis, the time series tested is considered nonstationary, and if one model rejects the null hypothesis, the time series is considered stationary.

### 3.2. Mode Identification Based on the VLSW-MADF Test

The traditional ADF test introduced in Section 3.1 can only test the stationarity of a single variable. The production data from actual industrial processes are multivariable. As a result, the ADF test cannot be directly applied to industrial process data. However, in pattern recognition, the work that must be carried out is to recognize a pattern according to the changing trend of each variable. If we can extract a single variable that can represent the fluctuation trend of the multivariable industrial data, we can carry out pattern recognition through a stationarity analysis of the single variable. The single variable that can achieve this effect is called the trend variable of the process. This paper proposes a method of using mean value processing to extract the trend variables of a given process. We find that when the process is in a stable mode, the mean value of each sampling point also remains relatively stable; when the process is in a transitional mode, the mean value of each sampling point likewise fluctuates. The trend variables of the process extracted by the mean processing method can reflect the change trend of the process data accurately. The method for extracting the trend variable is described in detail in the second half of this section.

However, the MADF test alone cannot realize mode identification for process data. If we use the MADF test directly on a whole dataset with multiple modes without distinguishing between them, we will obtain incorrect test results, which cannot be reflected when the process enters a new mode. Only after the process data are divided can mode identification be realized by the MADF test. The result of mode identification is closely related to the length of the selected partition. Therefore, this paper combines the MADF test with a variable-length sliding window and finally proposes the VLSW-MADF test for modal identification. The approximate framework of the method is as follows: First, a window of length H is used to divide the trend variables of the given data, and then the ADF test is used for rough mode identification. Rough mode identification can be used to roughly distinguish stable modes from transition modes. Then, a window of length L is used to divide the trend variables, and the ADF test is used for detailed mode identification. Detailed mode identification can be used to determine the beginning and end of a transition mode. Finally, mode identification is realized for the multimodal process.

We provide the user with a criterion to select the hyperparameters of the proposed offline method. The parameter L should be chosen to satisfy the length of the minimum transition mode of the current process. According to the experience of modeling multivariate statistical regression methods, the window data should be sampled at least 2–3 times more than the number of variables in order to achieve an effective statistical feature extraction. The parameter H should be chosen to satisfy the length of the minimum stable mode of the current process. Moreover, the window length H should be chosen to be at least two times the window length of L (H≥2L).

The above procedure is the VLSW-MADF test proposed in this paper. It is worth noting that there is no difference between the final modal recognition results obtained using mean processing before and after sliding window partitioning. However, when the mean value is used to divide the sliding window, the method needs to solve the mean value many times. In the VLSW-MADF test proposed in this paper, mean value processing is used before sliding window partitioning to reduce the number of required calculation steps. That is, a sliding window partition and an ADF test are directly carried out on the trend variables.

More detailed steps are as follows. It is assumed that we have multimodal process data $\;X={\left[x_{m}\left(1\right),x_{m}\left(2\right),\ldots ,x_{m}\left(n\right)\right]}^{T}\in {\mathbb{R}}^{n\times m}(n$ is the number of samples, and m is the number of variables). The mean value of the sample point data $x_{m}\left(n\right)={\left[\alpha_{n1},\alpha_{n2,\cdots ,}\alpha_{nm}\right]}^{T}\in {\mathbb{R}}^{m\times 1}$ is calculated to obtain:

(21) $${\bar{x}}_{m}\left(n\right)=\frac{\sum_{i=1}^{m}\alpha_{ni}}{m}$$

By summarizing the results of Equation (21), the trend variable of the process is finally obtained as follows:

(22) $$\bar{X}=\left[{\bar{x}}_{m}\left(1\right),{\bar{x}}_{m}\left(2\right),\ldots ,{\bar{x}}_{m}\left(n\right)\right]\in {\mathbb{R}}^{1\times n}$$

Next, rough mode identification is performed: $\bar{X}$ is segmented along the sampling direction using a sliding window H. In this paper, we choose the window length based on the aforementioned criterion and the characteristics of the actual process. In the two numerical simulation cases of this paper, the window length of L is determined to be 50 and the window length of H is determined to be 100. After cutting, we obtain a series of windows: ${\bar{X}}_{1}=\left[{\bar{x}}_{1},{\bar{x}}_{2},\ldots ,{\bar{x}}_{H}\right]\in {\mathbb{R}}^{1\times H},\;{\bar{X}}_{2}=\left[{\bar{x}}_{H+1},{\bar{x}}_{H+2},\ldots ,{\bar{x}}_{2H}\right]\in {\mathbb{R}}^{1\times H}$…. The ADF test in Section 3.1 is used to test the stationarity of the data in each window. Finally, a stationarity matrix is obtained as follows:

(23) $$H^{*}=\left[h_{1},h_{2},\ldots h_{H}\right]$$

where $h=\left\{\begin{array}{c} 0\;\mathrm{Data}\;\mathrm{are}\;\mathrm{in}\;a\;\mathrm{nonstationary}\;\mathrm{state}\\ 1\;\mathrm{Data}\;\mathrm{are}\;\mathrm{in}\;a\;\mathrm{stable}\;\mathrm{state} \end{array}\right.$.

The nonstationarity window data correspond to processes in a transition mode, while the stationarity window data correspond to processes in a steady mode. The resulting stationarity matrix $H^{*}$ shows the result of rough mode identification for a multimodal process. However, at this point, we can only obtain a rough idea of whether the process corresponding to each segment of data is in a stable mode or a transitional mode. To achieve pattern recognition, it is necessary to further determine the positions of the beginning and end of each mode. Therefore, detailed mode identification is also needed.

Detailed mode identification: Based on the results of rough mode identification, for the previous window entering the nonstationary state to the next window ending the nonstationary state mode, the shorter window L is used for pattern recognition and stability testing. For example, assuming that the continuous $\left(p-q+1\right)$ values from $h_{p}$ to $h_{q}$ in the stationary matrix $H^{*}$ are all zero, the sample dataset that needs to be reidentified and retested is ${\bar{X}}_{new}=\left[{\bar{X}}_{p-1},{\bar{X}}_{p},\ldots ,{\bar{X}}_{q},{\bar{X}}_{q+1}\right]\in {\mathbb{R}}^{1\times \left(p-q+3\right)}$.

In this step, we need to choose a shorter window length L than H based on the aforementioned criterion and the characteristics of the actual process. We choose the window length L=50. Similar to the matrix obtained via the previous steps of rough mode identification, the final stationarity matrix is as follows:

(24) $$L^{*}=\left[h_{l1},h_{l2},\ldots h_{lL}\right]$$

**Figure 1:**
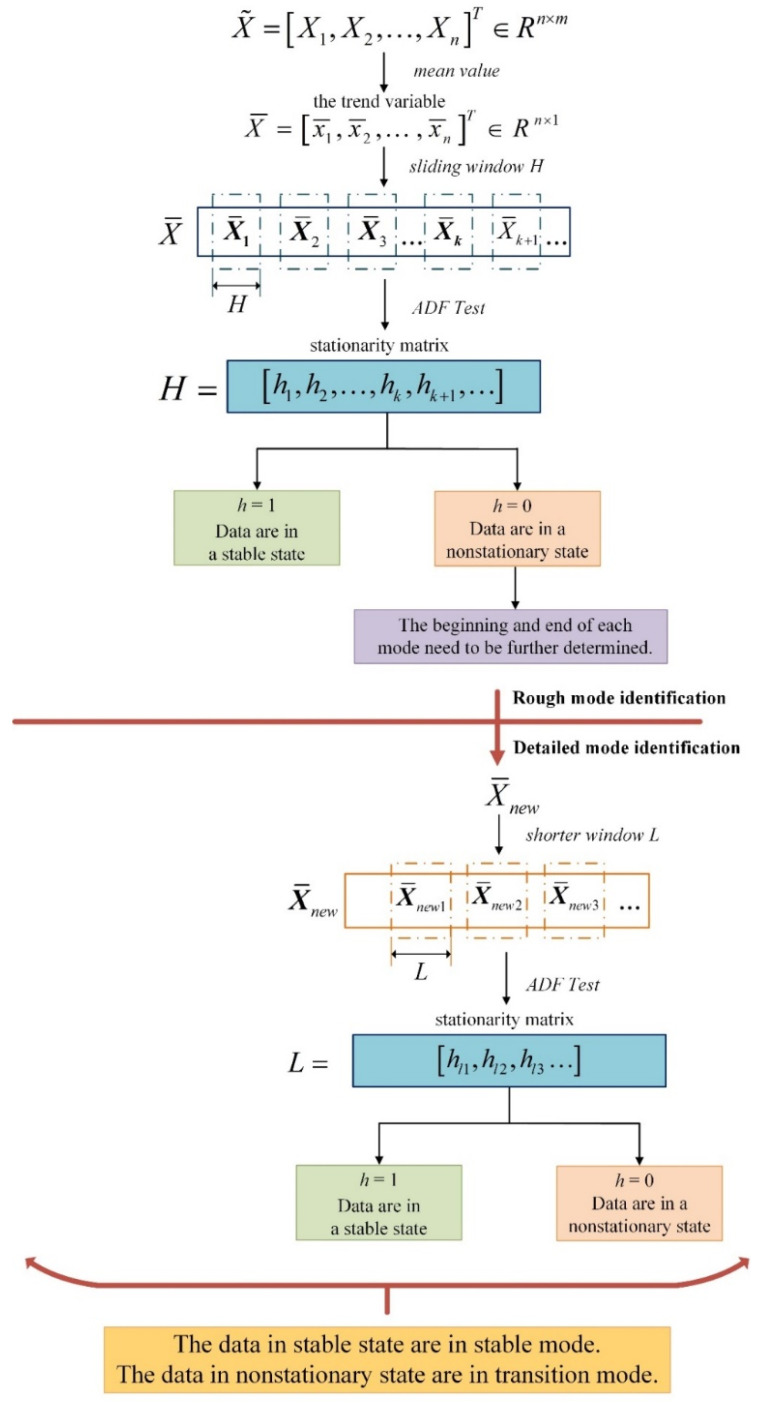
Illustration of the detailed steps of the VLSW-MADF test.

The starting point of the transition mode can be judged more accurately by matrix $L^{*}$ than by $\;H^{*}$. By analogy, the other transition modes in the rough pattern recognition results are determined in the same way, and finally, pattern recognition is realized for the multimodal process. The detailed steps of the VLSW-MADF test are shown in Figure 1.

### 3.3. Offline Modeling

The main idea of this method is to first identify a mode and then model it separately. In the offline modeling stage, the transition modes and stable modes obtained after pattern recognition should be modeled individually. In the second section, we declared that the process monitoring method used in this paper is DLPPCA. This method performs well on dynamic data and can accurately model transition modes with large variation ranges and strong dynamics. Although the stable mode means most of the time that the process data of that time series fluctuate around a stable central level, we still have to consider the serial correlation of the stable mode process data. Therefore, DLPPCA is equally applicable to offline modeling for both stable and transition modes. Therefore, this paper uses DLPPCA to model transition modes and stable modes separately based on mode identification. Algorithm 1 shows the offline modeling phase algorithm.
**Algorithm 1.** Offline modeling phase
Step 1: Input multimodal process data $X={\left[x_{m}\left(1\right),x_{m}\left(2\right),\ldots ,x_{m}\left(n\right)\right]}^{T}$;Step 2: Calculate trend variables $\bar{X}$ by (21) and (22);Step 3: Divide $\bar{X}$ through a window of length H, obtaining $H^{*}$ through ADF test;Step 4: Further divide the shorter window L into ${\bar{X}}_{new},$ obtaining $L^{*}$ through ADF test; Step 5: Obtain $X_{t1},X_{t2},\cdots$ by step 3 and step 4, and model them separately using DLPPCA, saving $T_{\alpha t}{}^{2}$ and $\;SPE_{\alpha t}$;Step 6: Similar to Step 5, obtain $X_{s1},X_{s2},\cdots$ by step 3 and step 4, and model them separately using DLPPCA, saving $T_{\alpha s}{}^{2}$ and $SPE_{\alpha s}$.

The specific steps are as follows:

Step 1: We acquire multimodal process data $X={\left[x_{m}\left(1\right),x_{m}\left(2\right),\ldots ,x_{m}\left(n\right)\right]}^{T}\in {\mathbb{R}}^{n\times m}$.

Step 2: The mean value of the training dataset X is calculated to obtain the trend variables of the process $\bar{X}=\left[{\bar{x}}_{m}\left(1\right),{\bar{x}}_{m}\left(2\right),\ldots ,{\bar{x}}_{m}\left(n\right)\right]\in {\mathbb{R}}^{1\times n}$.

Step 3: The VLSW-MADF test is used to test the stability of $\bar{X},$ determine the starting position of each mode, and complete the pattern recognition task.

Step 4: According to the results of mode division obtained in the previous step, the training data X are divided into several subsegments. The stable mode subsegments are $X_{s1},X_{s2},\cdots ,$ and the transition mode subsegments are $X_{t1},X_{t2},\cdots$.

Step 5: DLPPCA is used to model each transition mode subblock. Taking subsegment $X_{t1}$ as an example, according to the content in the first section, the confidence limit $T_{\alpha t1}{}^{2}$ and $\;SPE_{\alpha t1}$ can be obtained by using DLPPCA to model $X_{t1},$ and the final feature extraction result $T_{t1}$ can be obtained. This feature extraction result is also called the matching matrix. This matching matrix and the confidence limit are both saved.

**Figure 2:**
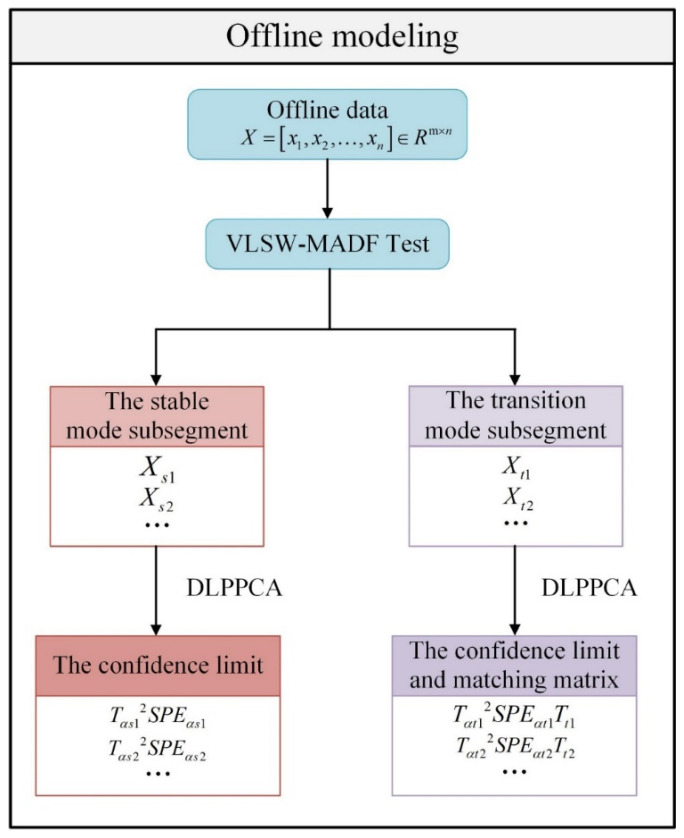
Flowchart of the offline modeling.

Step 6: Similar to Step 5, DLPPCA is also used to model the stable mode subblocks. Taking subsegment $X_{s1}$ as an example, DLPPCA is used to model and obtain the confidence limit $T_{\alpha s1}{}^{2}\;$ and $\;SPE_{\alpha s1}\;$. In the subsequent online mode identification step, it is not necessary to use the matching matrices of the stable modes, so only the confidence limit needs to be saved here. A flowchart of the offline modeling is shown in Figure 2.

It is worth noting that this paper only takes a portion of a process dataset as an example to illustrate the steps of offline mode identification and modeling. However, in practical applications, to model all the modes offline, it is often necessary to identify and model multiple segments of process data. In particular, a transition mode is assumed to contain a stable mode A and stable mode B. The transition process from stable mode A to stable mode B (transition mode AB) and the transition process from stable mode B to stable mode A (transition mode BA) are two different transition modes, and the change trends of their related characteristics are also different, so it is necessary to establish transition models for these two transition modes separately. In other words, if we have stable modes A and B, then A and B satisfy $B\leq A\left(A-1\right)$.

## 4. Online Mode Identification and Monitoring Algorithm

In the last section, offline mode identification and modeling were completed. When conducting online monitoring for multimodal processes, it is also necessary to identify the current online process data. Only by determining which mode the current process belongs to can the appropriate offline model for subsequent online monitoring be selected. If the judgment is wrong, it may result in false alarms. Therefore, when online monitoring is performed, it is necessary to carry out mode identification first and then statistical monitoring.

For online mode identification, researchers have proposed several methods, such as the minimum SPE principle, which involves traversing all models and selecting the corresponding model with the lowest SPE. Another approach is the probability monitoring method, in which the online samples come from each process with a certain probability, and all offline models are used for joint detection with a certain probability.

However, all offline models need to be considered in the above methods. When there are too many modes in the examined process, the number of calculations is too large. When the process corresponding to the online data first enters the transition mode, the amount of data is small, and the data characteristics are different from those of the whole transition dataset. If the offline model derived from the whole transition dataset is used to match the online data, mode identification errors easily occur.

Therefore, considering the above problems, a new online mode identification method is proposed. The method proposed in this paper does not need to identify all online data but instead discusses them in different situations, and mode identification is performed only in certain situations. The proposed mode identification method is based on the calculation of matching values. An offline matching matrix with the same sample length as that of the online dataset is used for mode identification instead of using the whole transition dataset for matching, thereby improving the accuracy of the method.

It should be noted that since the current sampling time selected for online operation is k, reliable and accurate conclusions cannot be obtained if the online modal recognition process depends only on the results of one sample point at time k. Therefore, online mode identification is performed by combining the recognition results of ω consecutive online sampling data, that is, from the $\left(k-\omega +1\right)$ sample to the kth sample. Algorithm 2 shows the online monitoring phase algorithm. Based on the above premises, the detailed steps of the online monitoring method proposed in this paper are as follows (the proposed online mode identification method is used in Step 4).
**Algorithm 2.** Online monitoring phase.
Step 1: Input online process data $X_{online}$;Step 2: Determine the mode of the starting phase by minimum SPE;Step 3: Monitor the current continuous ω data from $\left(k-\omega +1\right)$ to k using an offline model corresponding to $\left(k-\omega \right)$ time data;

    Situation 1: Below the control limit.
        The current process mode is the same as the previous one.
    Situation 2: Exceeding the control limit.
        Situation 2.1: The current process has a fault situation.
        Situation 2.2: The current process enters a new mode.
          Situation 2.2.1: The process is in transition mode at the previous moment.
          Situation 2.2.2: The process is in stable mode at the previous moment.Step 4: For situation 2.2.2, calculate matching value $m_{i}$ for online modal recognition.Step 5: Use the model determined in step 4 to remonitor. If it is below the control limits, the currently selected model matches the actual mode. If it exceeds the control limits, a fault has occurred.

Step 1: Determining the model for the starting stage.

For the initial phase of a process, since there are no data from the sample points at the previous moment to use as references, the corresponding model for the starting phase needs to be determined. Here, the minimum SPE principle is used to determine that for a data segment with a starting length of ω; the online data are monitored in turn using known historically stable modes. The model with the lowest SPE for online samples is selected for monitoring.

Step 2: Trial monitoring of online process data:

When there are $\left(k-\omega +1\right)$ consecutive ω data points to k, the offline model from the previous moment is fully utilized for detection. The current continuous ω (ω > 10) data from $\left(k-\omega +1\right)$ to k are monitored using an offline model corresponding to $\left(k-\omega \right)$ time data.

Step 3: Analyzing the monitoring test results.

There are several possibilities for monitoring the test results obtained in Step 2. If the current process data statistic is below the control limit, it means that the process data ω and (*k* − *ω*) correspond to the same mode of the process. If the current process data statistic exceeds the control limit, the mode changes at the time when the control limit is exceeded. There are two possibilities for change.

1. The current process has a fault situation;
2. The current process enters a new mode. There are also two possibilities for entering a new modal process, from transition mode to stable mode or from stable mode to transition mode.

It should be noted that to avoid false alarms, this paper considers that a process is abnormal only when a continuous number of samples ($\geq \frac{\omega }{2}$) are beyond the control limit and do not depend only on the identification result of a sampling point at time t.

Step 4: Online modal identification.

If the current process data statistics are beyond the confidence limit, it is necessary to judge whether the current process is having a fault or enters a new mode.

First, we assume that the current data enter a new mode.

3. If the process was in the transition mode at the previous moment (*k* − *ω*), the current process enters the stable mode corresponding to the transition mode. This stable mode is selected as the monitoring mode and no mode identification is required;
4. If the previous moment (*k* − *ω*) process is in a stable mode, the current process enters into a transition mode that bridges with this stable mode. Modal identification is required. However, only the transition modes that articulate that stable mode need to be selected for modal identification, not all transition modes need to be selected.

In online mode identification, a method based on matching value calculation is presented in this paper.

Assume that all possible historical transition modes are $X_{t1},X_{t2},\cdots$. According to Section 3.3, we can obtain the matching matrix corresponding to the historical transition mode: $T_{t1},T_{t2},\cdots$. The online data are modeled by DLPPCA, and the online matching matrix is $T_{online}$. The historical matching matrix at this time comes from performing feature extraction on the whole transition dataset, and $T_{online}$ is derived only from the current ω data points. The characteristics of transition data are high volatility and a large change range. This means that $T_{online}$’s data features are different from those of $T_{t1},T_{t2},\cdots$. Additionally, direct matching is prone to errors. Therefore, to conduct matching accurately, short processing is performed for $T_{t1},T_{t2},\cdots$; that is, the original $T_{ti}\in {\mathbb{R}}^{d\times \left(n-l\right)}$ is truncated along the direction of the sampling point, and only the first ω column vectors are taken. Because the data needed for online mode identification are considered to have just entered the transition mode, it is reasonable to select the first ω column vectors of $T_{ti}$.

Next, the matching value $m_{i}$ between the matrices $T_{online}$ and $\tilde{T_{ti}}$ is calculated in turn. In this paper, the matching value $m_{i}$ is solved based on Euclidean distance, and the similarity of the two matrices is measured by calculating the sum of the distances between the corresponding column vectors in $T_{online}$ and $\tilde{T_{ti}}$. The smaller the distance, the smaller $m_{i}$ is. The specific procedure is as follows:

First, the Euclidean distance vector between $T_{online\;}$ and $\tilde{T_{ti}}\;$ is calculated as follows:

(25) $$D_{d}=\left[d_{1},d_{2}\cdots ,d_{j}\right]$$

where

(26) $$d_{j}=\sqrt{{\left(T_{onlinej}-\tilde{T_{tij}}\right)}^{T}\left(T_{onlinej}-\tilde{T_{tij}}\right)}$$

Here, $T_{onlinej}$ represents line j of $T_{online}$ and $\tilde{T_{tij}}$ represents line j of $\tilde{T_{ti}}$.

The elements of the Euclidean distance matrix D are summed to obtain the matching value $m_{i},$ as follows:

(27) $$m_{i}=d_{1}+d_{2}+\cdots +d_{j}$$

The transition mode corresponding to the minimum $\;m_{i}$ is selected as the monitoring model.

Step 5: Remonitoring.

**Figure 3:**
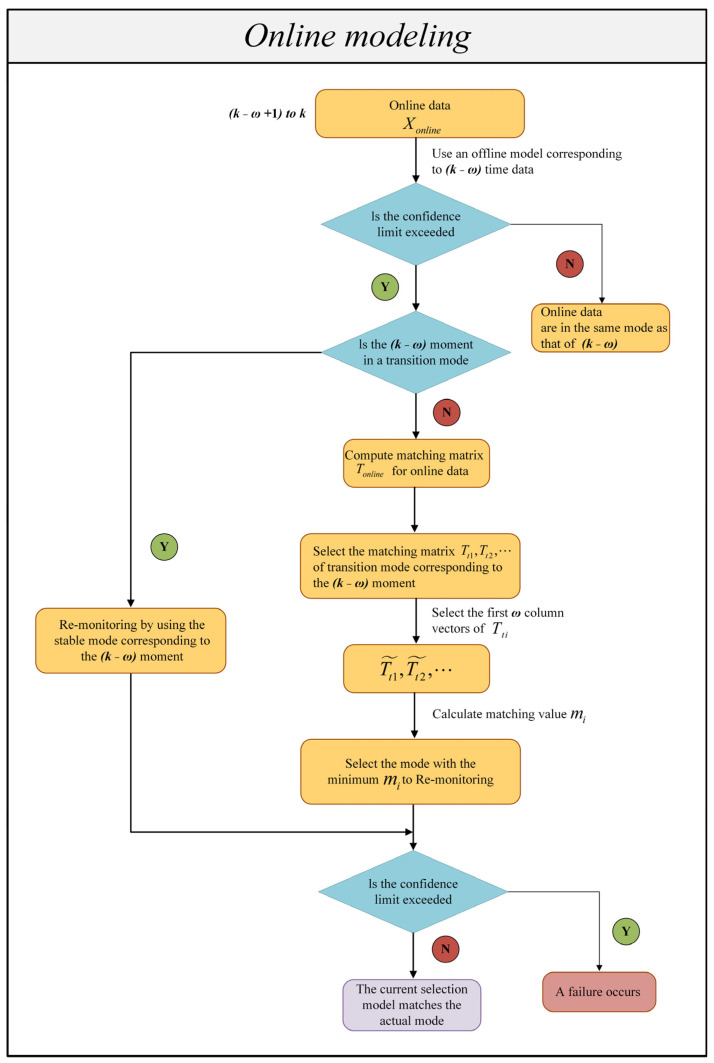
Flowchart of the online modeling.

The ω consecutive process data points from $\left(k-\omega +1\right)$ to k are monitored again using the monitoring model determined in Step 4. The monitoring results are analyzed again. If the current process statistics are below the confidence limit, indicating that the current selection model matches the actual mode, the obtained model can continue to perform process monitoring. If the current data still exceed the confidence limit, a fault has occurred. A flowchart of the online mode identification and monitoring algorithm is shown in Figure 3. To avoid confusion among the many symbols, we have created a nomenclature, as shown in Nomenclature Section.

## 5. Application and Results

In the previous section, we showed the proposed method in detail. In this section, we will use two numerical simulation cases to verify the effectiveness of our proposed method. First, the first numerical simulation case was carried out based on the TE process. We generated multimodal data based on normal operating conditions by adjusting the operating points of the TE process. Second, the case of the second numerical simulation was carried out based on data from a power plant generating unit. We also simulated a multimodal process data. The validity and feasibility of the methods presented in this paper are verified from the following four perspectives:

1. The presented offline mode identification method based on the VLSW-MADF test is accurate and feasible;
2. The online mode identification method proposed in this paper is accurate and feasible;
3. Transition modes are more accurately modeled and monitored using DLPPCA than with other approaches;
4. Modeling stable modes and transition modes separately can improve the accuracy of online monitoring.

For the proposed method, the fault detection rate (FDR), false alarm rate (FAR), missed alarm rate (MAR), and detection delay (DD) are mainly considered to evaluate the method’s performance. These metrics are applied to quantify the method performance in the two subsequent numerical simulation cases. False alarm rate (FAR) measures the probability of false alarms, and a false alarm is an indication of a fault when a fault has not occurred. Fault detection rate (FDR) measures the probability of successful fault detection, and successful fault detection is an indication of a fault when a fault has occurred. Missing alarm rate (MAR) measures the probability of a missed alarm, which is when a fault occurs but is not detected. Detection delay (DD) is the time period between the start of a fault and the time of the detection. It is expected that a larger value for the FDR indicator is better. Smaller values for the remaining three indicators are better. The formulae for calculating the FDR, FAR, and MAR indicators are as follows.

(28) $$\mathrm{FDR}=\frac{\;\mathrm{number}\;\mathrm{of}\;\mathrm{samples}\;\left(I>I_{\mathrm{CL}}|\mathrm{fault}\right)}{\;\mathrm{total}\;\mathrm{samples}\;(\mathrm{fault})}\times 100\%$$

(29) $$\mathrm{FAR}=\frac{\;\mathrm{number}\;\mathrm{of}\;\mathrm{samples}\;\left(I>I_{\mathrm{CL}}|\mathrm{fault}\;-\;\mathrm{free}\right)}{\;\mathrm{total}\;\mathrm{samples}\;(\mathrm{fault}\;-\;\mathrm{free})}\times 100\%$$

(30) $$\mathrm{MAR}=\frac{\;\mathrm{number}\;\mathrm{of}\;\mathrm{samples}\;\left(I\leq I_{\mathrm{CL}}|\mathrm{fault}\right)}{\;\mathrm{total}\;\mathrm{samples}\;(\mathrm{fault})}\times 100\%$$

where I represents the current data statistic value and $I_{\mathrm{CL}}$ represents the control limit. $I=\left\{T^{2},SPE\right\}$.

### 5.1. TE Process

A TE process is a simulation based on a real industrial process [45,46,47]. The operating points of a TE process can be adjusted to meet production requirements when generating multimodal data. This paper describes a 160 h multimodal process; the values of the Production Setpoint, Sep Level Setpoint, and Steam Valve Position are changed at the 50th hour so that the TE process transitions from stable mode A to stable mode B. At 90 h, the values of the production setpoint, sep level setpoint, steam valve position, mole%g setpoint, and yA setpoint are changed again so that the TE process transitions from stable mode B to stable mode C.

**Figure 4:**
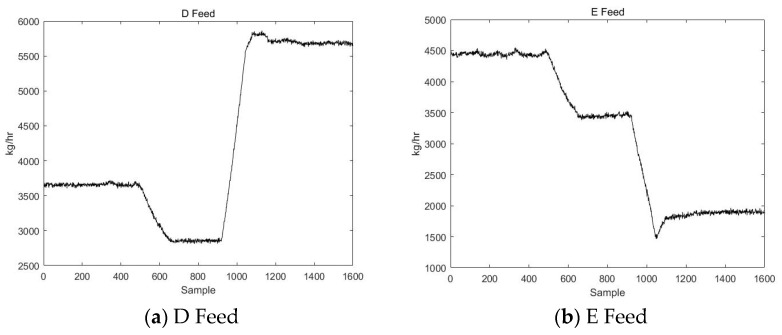

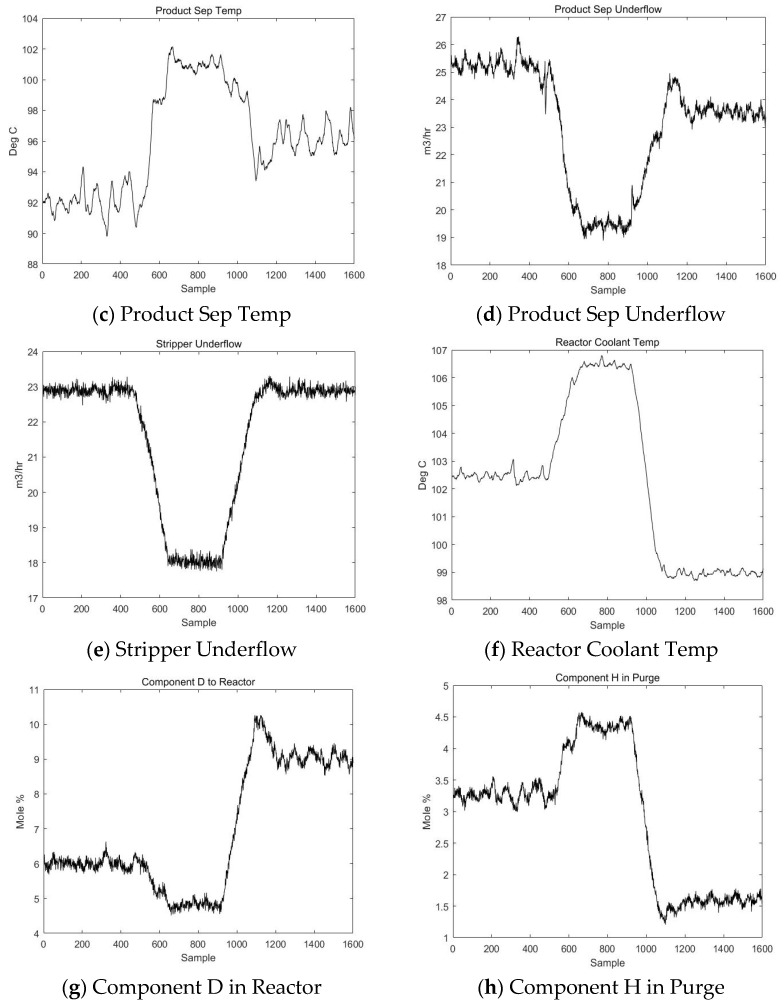
The change curves of the 8 variables of the simulation data.

Finally, multimodal process data were obtained, with a total of 1600 sample points. This process includes the stable mode A, stable mode B, stable mode C, transition mode AB, and transition mode BC. There are 53 variables in the TE process. Eight process continuous variables are selected to validate the proposed method. These eight variables are shown in the following Table 1. The change curves of the eight variables of the simulation data under normal working conditions are shown in Figure 4.

**Figure 5:**
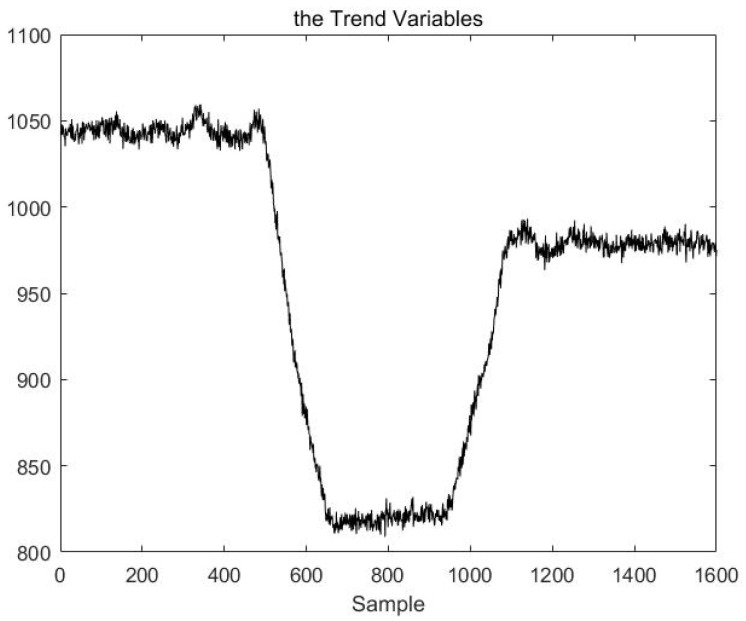
The change curve for this trend variable.

The multimodal process dataset consisting of these eight variables is named $X_{train}$. First, the segment data are identified based on the VLSW-MADF test. The trend variable $\bar{X}$ is derived from Formulas (21) and (22). The change curve for this trend variable is shown in Figure 5. Notably, the trend of $\bar{X}$ coincides with the pattern change trend of the original process; the pattern changes in the 50th and 90th hours and transitions to a new mode each time. This indicates that the variable $\bar{X}$ can represent the trend of multivariable process data.

After $\bar{X}$ is obtained, rough mode identification is performed using a window with a length of H=100, resulting in the following stationarity matrix:

$$H=\left[1,1,1,1,1,0,0,1,1,0,0,1,1,1,1,1\right]$$

It can be seen from the matrix that the window ${\bar{X}}_{1}-{\bar{X}}_{5}$ is in a stable mode. Window ${\bar{X}}_{6}-{\bar{X}}_{7}$ is nonstationary and enters a transition mode. Window ${\bar{X}}_{8}-{\bar{X}}_{9\;}$ enters a stable mode. Window ${\bar{X}}_{10}-{\bar{X}}_{11}$ enters a transition mode again. The final window ${\bar{X}}_{12}-{\bar{X}}_{16}$ remains in a stable mode. In order to highlight the details in the mode transitions, the next step requires more detailed mode identification to determine the exact starting position of the transition modes. Two small stationarity matrices are obtained by dividing the data of windows ${\bar{X}}_{5}-{\bar{X}}_{8}$ and ${\bar{X}}_{9}-{\bar{X}}_{12}$ using a shorter window L=50. The small stationary matrices are shown below:

$$L_{1}=\left[1,1,0,0,0,1,1,1\right]\;L_{2}=\left[1,1,1,0,0,0,1,1\right]$$

**Figure 6:**
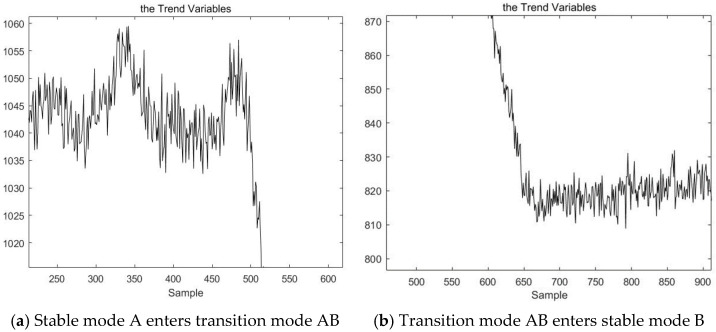

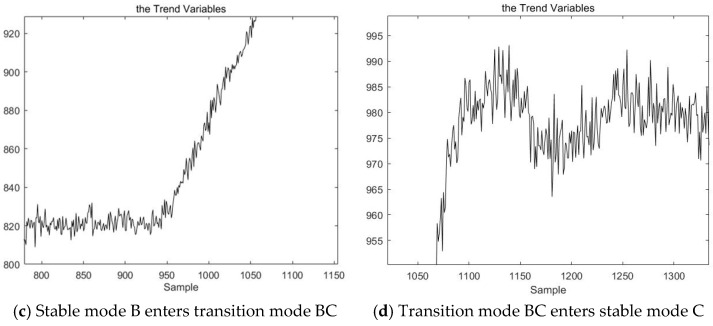
The local magnifications of the trend variable.

Finally, results are obtained based on the VLSW-MADF test. The process of data points 1–500 is in stable mode A. The process of data points 500–650 is in transition mode AB. the process of data points 650–950 is in stable mode B. the process of data points 950–1100 is in transition mode BC. Finally, the process of data points 1100–1600 is in stable mode C. These results are consistent with the actual situation and can be explained with the trend variable $\bar{X}$. Figure 6 is the local magnifications of the trend variable $\bar{X}$ for demonstrating the correctness of the results of mode identification based on the VLSW-MADF test.

Next, based on the modal identification results, stable modes A, B, and C and transition modes AB and BC are modeled using DLPPCA, and the confidence limits and matching matrices of each mode are saved. It is important to note that only a portion of the full dataset is presented here. However, other forms of multimodal data need to be identified and modeled. Finally, the confidence limits and matching matrices of stable modes A, B, and C and transition modes AB, BC, AC, BA, CB, and CA are obtained. In the online monitoring phase, this paper first monitors a section of normal operating process data from stable mode B to stable mode C online. The test data contain a total of 1000 sample points, and the process enters transition mode BC at around the 500th sample point, exits transition mode at around the 700th sample point, and finally enters stable mode C.

**Figure 7:**
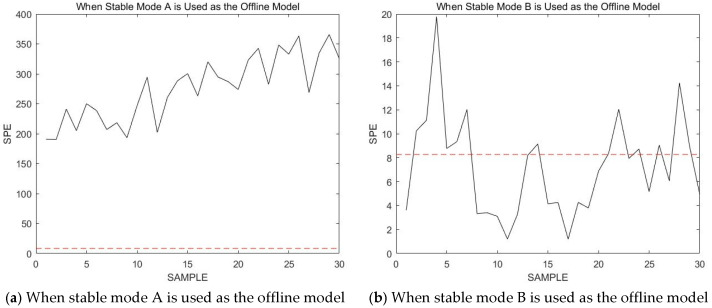

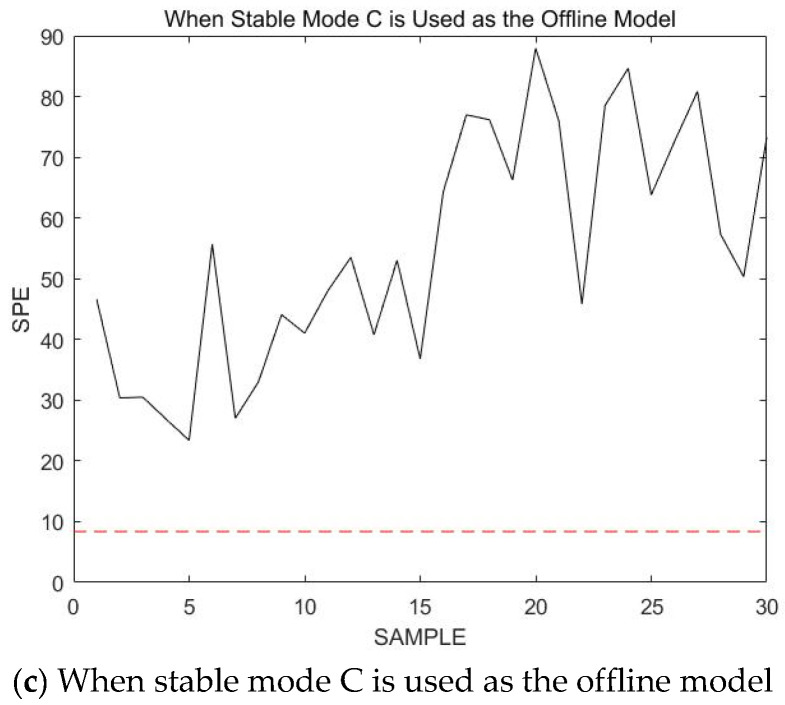
The obtained results in terms of the SPE statistics.

This test dataset $X_{test}$ is used to verify the correctness of the online modal identification method proposed in this paper. As seen in Section 4, there are no previous sample data points available for reference at the beginning of the process. Therefore, the online data are monitored using known stable modes A, B, and C as offline models; that is, 30 consecutive data points from the first sample are monitored online. The obtained results in terms of the SPE statistics are shown in Figure 7.

The red dashed line in the figure represents the confidence limit. Notably, the SPE statistic is the smallest when using stable mode B to detect online process data. From this observation, it is determined that the production process is in stable mode B. This conclusion is consistent with the actual situation. The online process data are then continuously monitored using stable mode B. In subsequent monitoring steps, most of the online data statistics are below the confidence limit, and these online data statistics only occasionally exceed the confidence limit. However, it has been declared previously that a failure or a new mode is only considered if several consecutive sampling points exceed the confidence limit.

**Figure 8:**
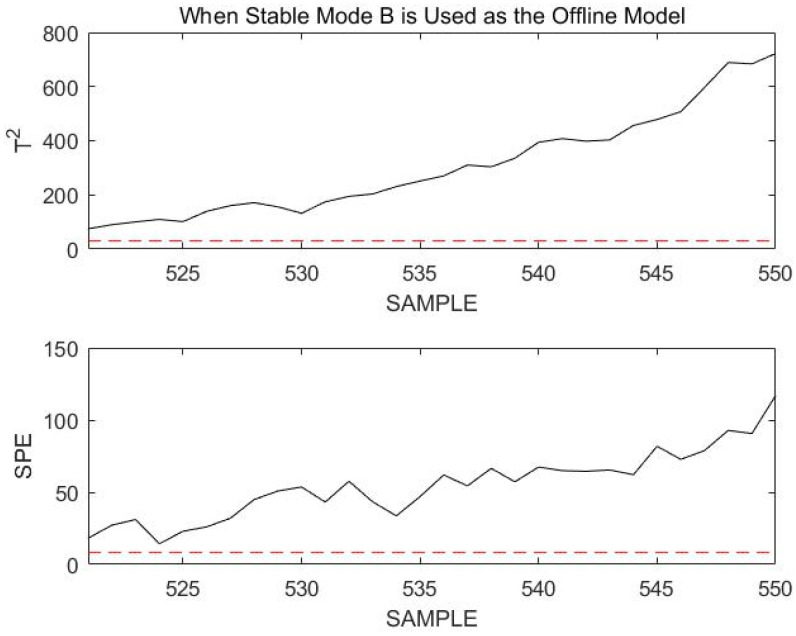
Monitoring 521st to 550th sample points using stable mode B as an offline model.

When the 521st sampling point to the 550th sampling point is monitored, these 30 consecutive sampling points are beyond the confidence limit, as shown in Figure 8.

**Figure 9:**
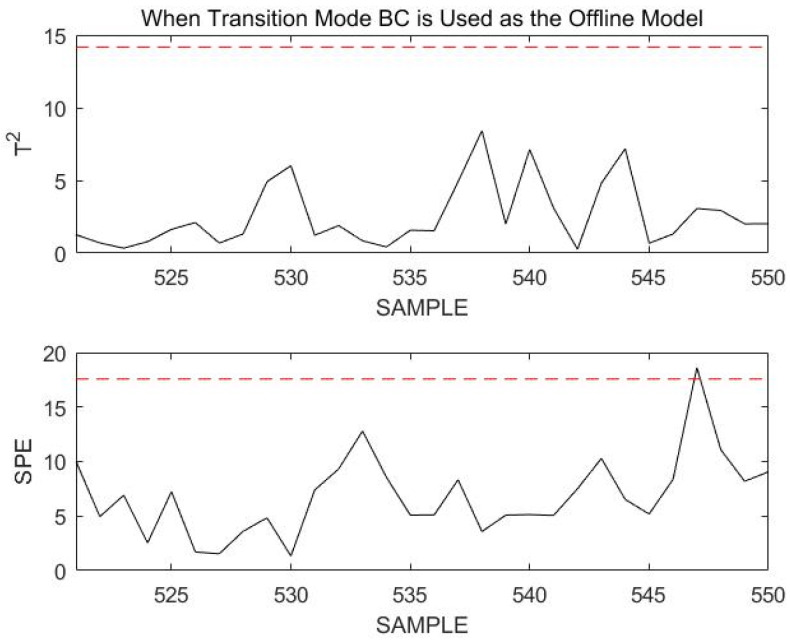
Monitoring 521st to 550th sample points using transition mode BC as an offline model.

At this point, we consider the current process to have transitioned to a new operating mode or to have a fault. The steps in Section 4 are now followed. First, assume that the current process transitions to a new operation mode. Since the process at the previous time is in stable mode B, the current process of this section must be in one of the transition modes connected to B. Therefore, transition mode BA, transition mode BC, and the current process must be selected to match the current mode. According to Step 4 in Section 4, the matching value between the online data and transition mode BA is $m_{BA}=21.98,$ and the matching value for transition mode BC is $m_{BC}=17.41$. According to these matching values, it is determined that the current process enter transition mode BC. Transition mode BC is used as the offline model to remonitor the current process data online, and the results are shown in Figure 9.

At this time, the online monitoring sample statistics are below the confidence limit. It is proven that the current process is in transition mode BC, which is consistent with the actual situation. The above simulation verifies the feasibility and correctness of the online mode identification method proposed in this paper that performs online monitoring and online modal identification on process data obtained under normal working conditions.

**Figure 10:**
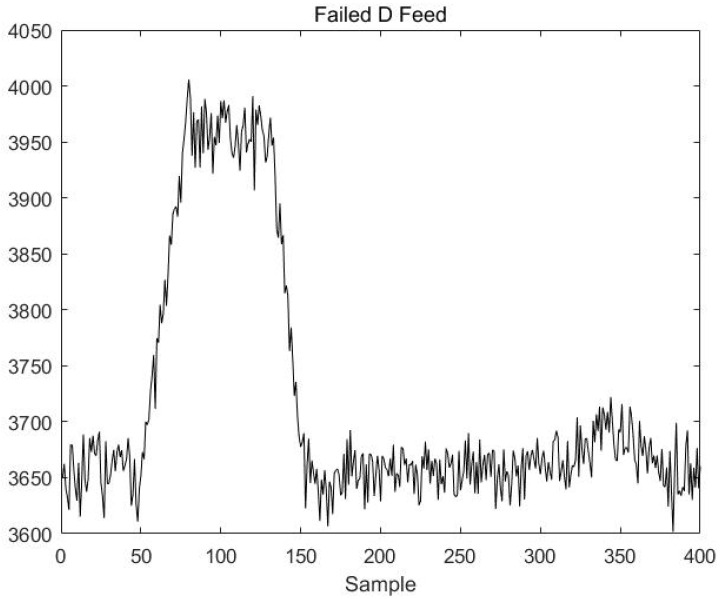
The change curve of D Feed.

The next step is to use a data segment in stable mode A where a fault has occurred online test data. The fault occurs in the variable D Feed; introducing a fault signal at the 51st sample point linearly increases the variable D Feed to simulate a progressively increasing fault. The increased value is maintained between the 80th and 130th sample points; starting at the 131st sample point, the value falls back to its normal level, and at the 150th sample point, the fault disappears. The change curve of D Feed is shown in Figure 10.

**Figure 11:**
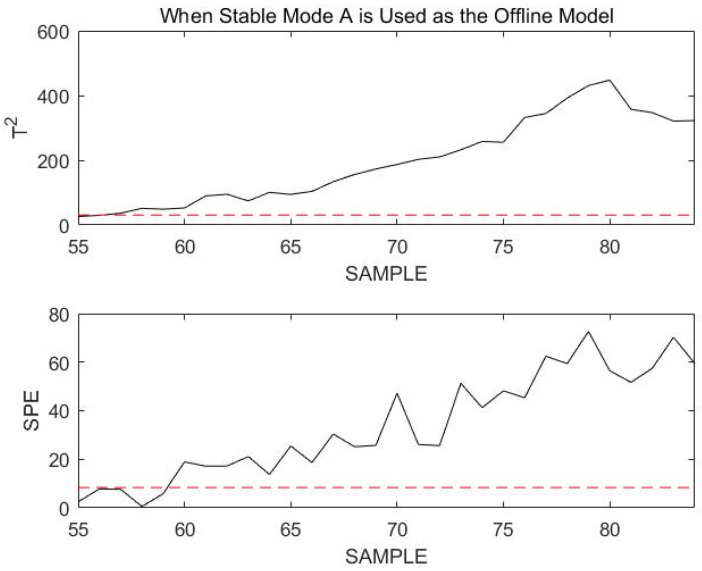
Monitoring 55th to 84th sample points using stable mode A as an offline model.

The simulation here omits steps such as determining the initial mode and begins directly at the 55th sample point. Since the historical mode of the data at the previous moment is known to be stable mode A, stable mode A continues to be used for the online monitoring of 30 consecutive data points starting at the 55th sample point, as shown in Figure 11.

**Figure 12:**
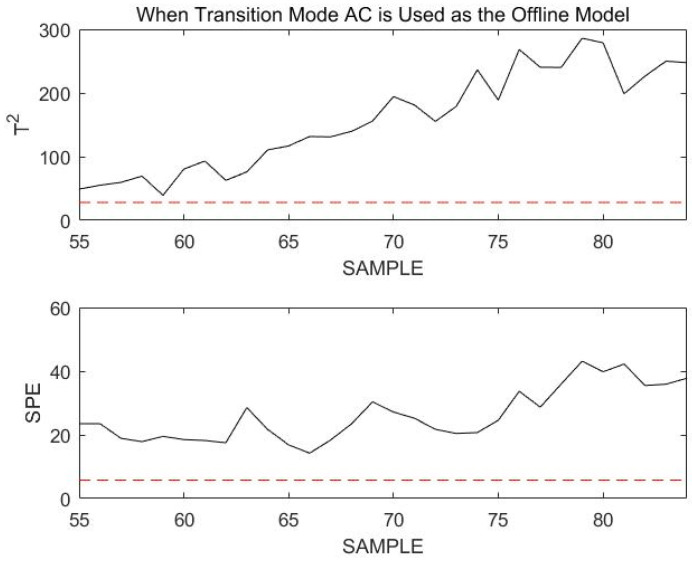
Monitoring 55th to 84th sample points using transition mode AC as an offline model.

As seen in the figure above, the $T^{2}$ and SPE statistics for the current data almost all exceed the confidence limit. As a result, the current process has faulted or entered a transitional mode. Assuming that the current process enters a transition mode, since the previous moment was stable mode A, the current process can only be in a transition mode joined to A: transition mode AB or transition mode AC. The procedure of Step 4 in Section 4 is continued to obtain the match between the online data and transition mode AB;$\;m_{AB}=41.05,$ and the matching value for transition mode AC is $m_{AC}=28.99$. From the matched values, transition mode AC is more likely to be the transition mode in which the online process is located. The online data are remonitored using transition mode AC, as shown in Figure 12.

Obviously, all data statistics exceed the confidence limit. This indicates that the current process does not enter transition mode AC, but is faulted. Therefore, the current process data will continue to be monitored using stable mode A. To better illustrate the effectiveness of using stable mode A for the failure monitoring of online data, Figure 13 shows the results of online monitoring at sample points 51 to 200.

**Figure 13:**
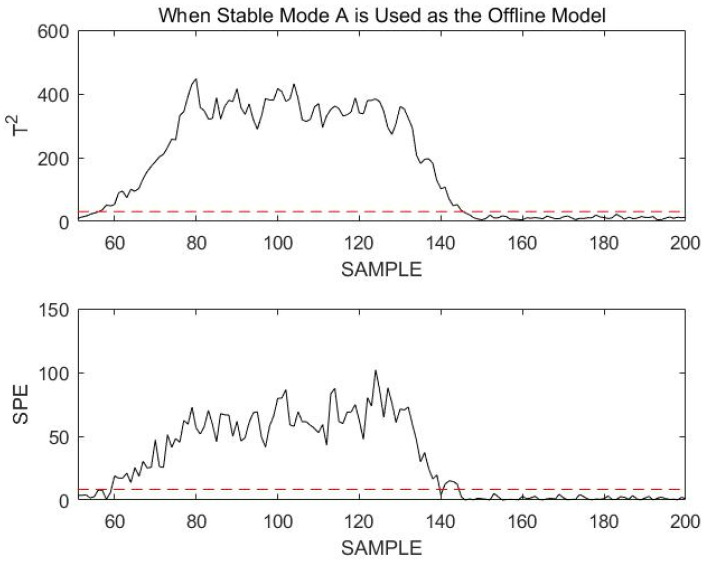
Monitoring 51st to 200th sample points using stable mode A as an offline model.

Figure 13 in the article shows the results of online monitoring based on the modal identification monitoring model. The fault can be clearly detected when $T^{2}$ and SPE exceed the control limits at 56 and 60 sampling times, respectively. The fault is introduced from the 51st sampling time. The monitoring statistics $T^{2}$ and SPE have a detection delay of 5 sampling times and 9 sampling times, respectively. In addition, the estimated fault end time differs from the real situation by only 4 sampling times, which indicates that the monitoring model DLPPCA based on modal identification can accurately locate the fault interval and has accurate monitoring results. In addition, we calculated FDR, FAR, MAR, and detection delay for the $T^{2}$ statistic and SPE statistic, as shown in Table 2.

The above simulation of the TE process data proves the following:

1. The correctness and feasibility of offline mode identification based on the VLSW-MADF test. The VLSW-MADF method proposed in this paper can accurately and quickly identify the mode of multimodal process.
2. The correctness and feasibility of the online mode identification method proposed in this paper. The online mode identification method proposed in this paper does not require all the online data to be modally identified and makes full use of the data from the previous moment for a case-by-case discussion. When a fault occurs or enters a transition mode, this method can accurately identify.

In Section 5.2, the validation simulation of the actual data from a power plant motor set will be continued to demonstrate the superiority of the DLPPCA method and the necessity of modeling stable modes and transition modes separately.

### 5.2. Power Plant Data Simulation

**Figure 14:**
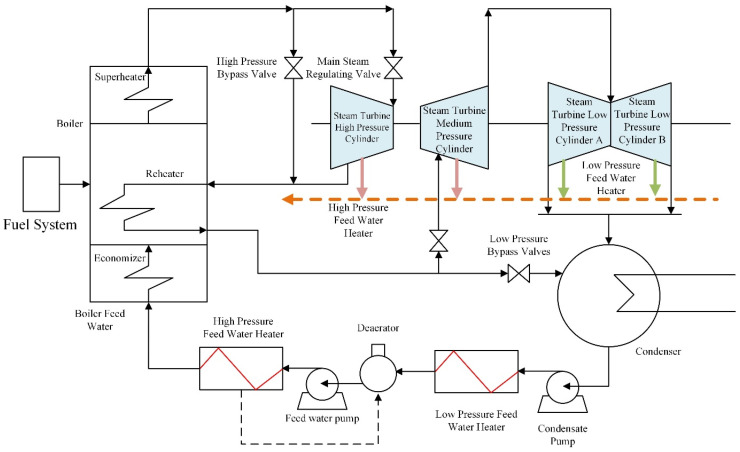
Schematic diagram of thermal power unit.

**Figure 15:**
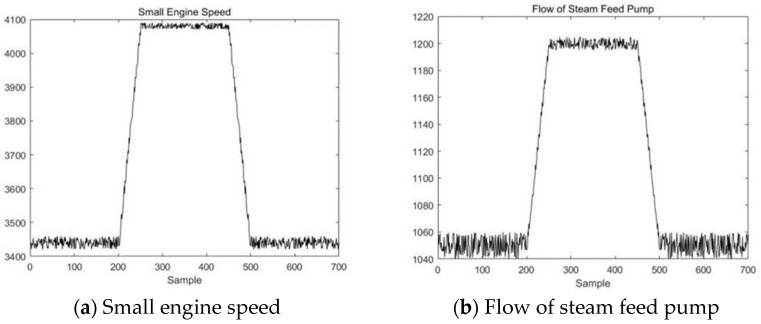

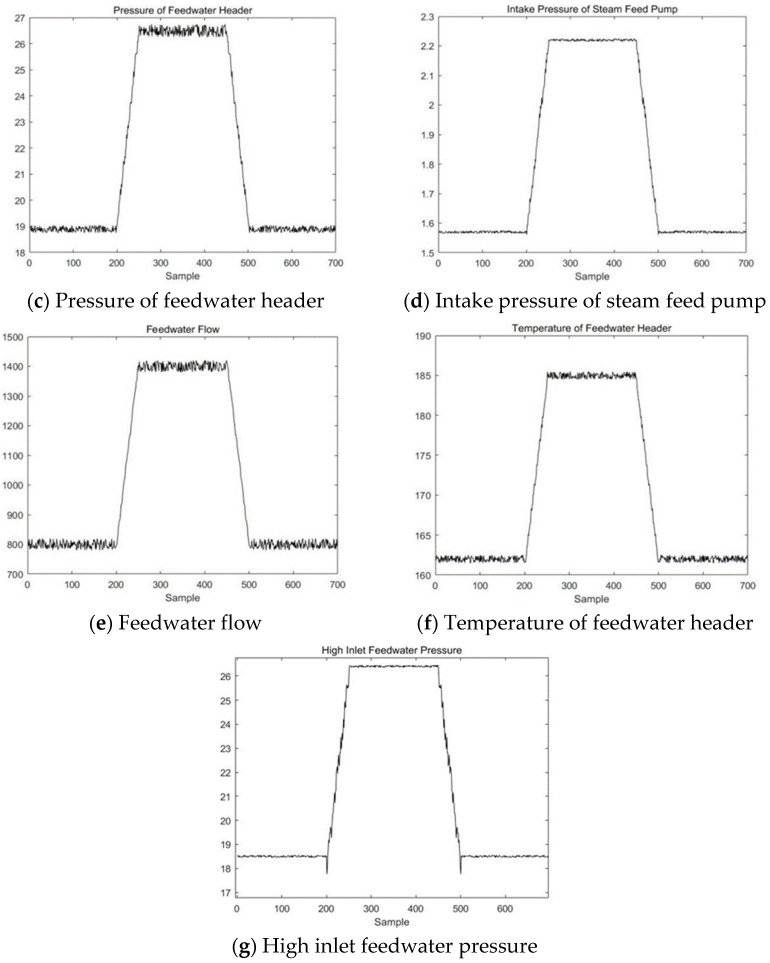
The change curves of the 7 variables of the simulation data.

In the simulation experiments in this section, relevant data from a 2 × 660 MW power plant are used. A steam feedwater pump system is selected as an example for simulation purposes. The schematic diagram of the thermal power unit is shown in Figure 14. The steam feedwater pump system contains seven variables, as shown in Table 3. The change curves of these seven variables are shown in Figure 15.

First, the VLSW-MADF method is used for offline modal identification of the multimodal process. It is determined that the process begins at the 200th sampling point, enters transition mode AB from stable mode A, and then enters a new stable mode (B) after 50 sampling points. At the 450th sample point, the process starts from stable mode B, enters transition mode BA, and then enters stable mode A after 50 sample points. After mode identification, the DLPPCA method is used to model stable mode A, stable mode B, transition mode AB, and transition mode BA. The confidence limits of each mode and the matching matrices of the transition modes are saved. Since this part of the procedure is the same as the simulation of the TE process in Section 5.1, it will not be repeated here.

**Figure 16:**
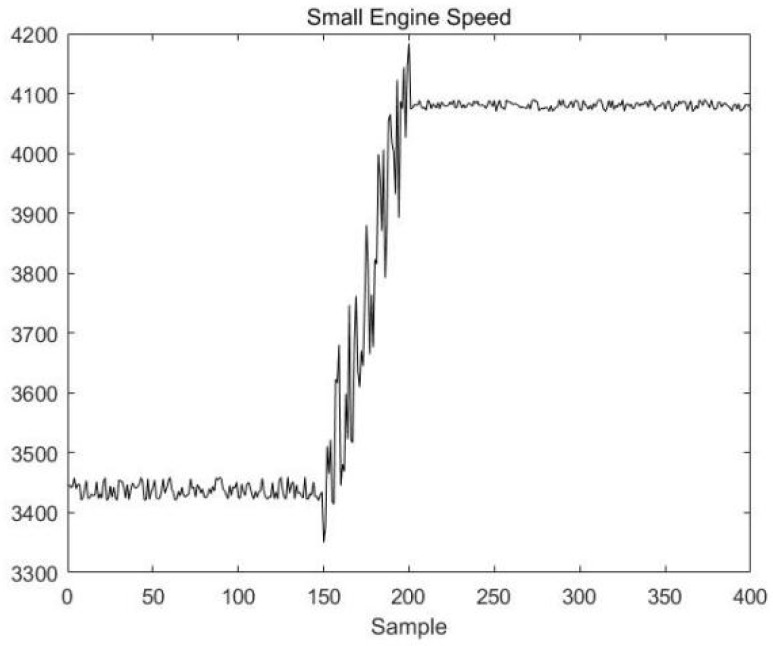
The change curve of the small engine speed.

To prove that DLPPCA performs well with dynamic transition data, fault data in each transition mode are used for online detection; there are 400 sample points in the test dataset, and the transition from stable mode A to stable mode B begins gradually at 150th sample point. A fault signal is introduced to transition mode AB of the variable “small engine speed” to simulate a noise fault during the transition. The variable change curve is shown in Figure 16.

**Figure 17:**
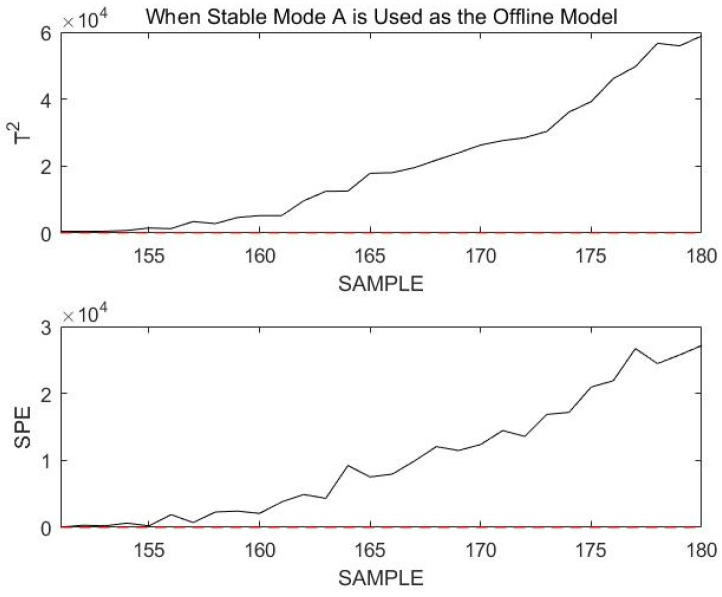
Monitoring 151st to 180th sample points using stable mode A as an offline model.

Ignoring the initial mode matching process, the online monitoring effect is demonstrated for 30 consecutive sample points, starting at sample point 151. Since at the previous moment the process was in stable mode A, the online process data are monitored using stable mode A first, and the results are in Figure 17.

**Figure 18:**
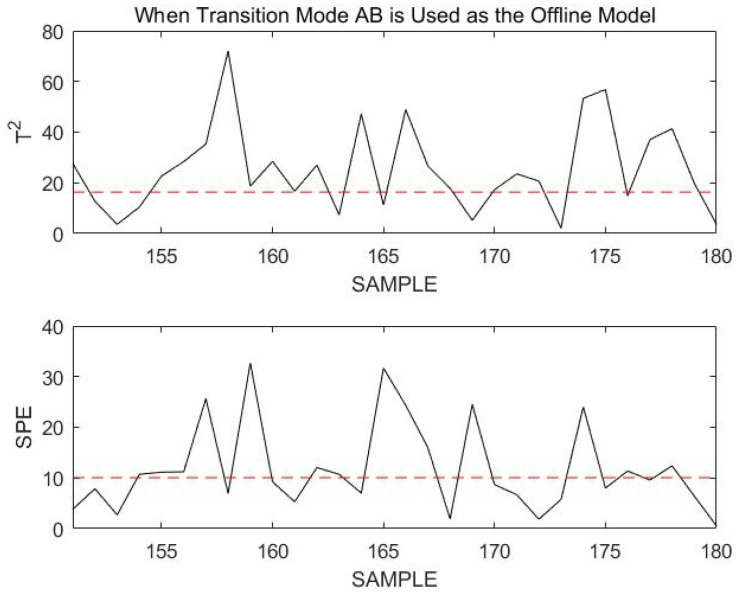
Monitoring 151st to 180th sample points using transition mode AB as an offline model.

Obviously, all sample points exceed the confidence limit. Assuming that the current process enters a new mode since it was in stable mode A at the previous moment, the current mode may only be in transition mode AB. The online data are then remonitored using transition mode AB, as shown in Figure 18.

**Figure 19:**
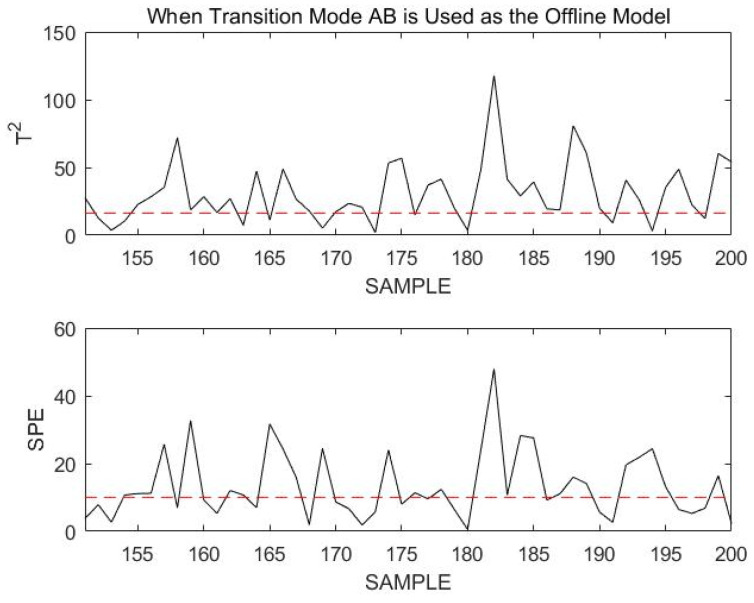
Monitoring 151st to 200th sample points using transition mode AB as an offline model.

If the current process mode is in transition mode AB, the statistics of the online data should be below the confidence limit when using transition mode AB for online monitoring. However, at this time, the confidence limit of the online data almost completely exceeds the confidence limit. This indicates that the online data are not in transition mode AB, but have a fault. Sample points 150 to 200 are monitored using transition mode AB, as shown in Figure 19.

From the monitoring results shown in the figure above, it can be seen that most of the sample points exceed the confidence limit when using transition mode AB to monitor the online process data. Although the sample points did not exceed the confidence limit by much, the occurrence of a fault was also identified.

These monitoring results are due to the nature of a fault itself. Transition mode data are dynamic, and the fault that occurs is that noise signals are added based on the original change trend, thereby increasing the fluctuation amplitude of the transition data. Therefore, it is difficult to monitor such faults. However, the DLPPCA method can still accurately model and monitor such transition data online. To illustrate the excellence of the DLPPCA method, a comparison is made between it, the DPCA method, and the LPPCA method without dynamic expansion. Only the modeling and monitoring methods are replaced in the comparison; all other steps remain the same.

**Figure 20:**
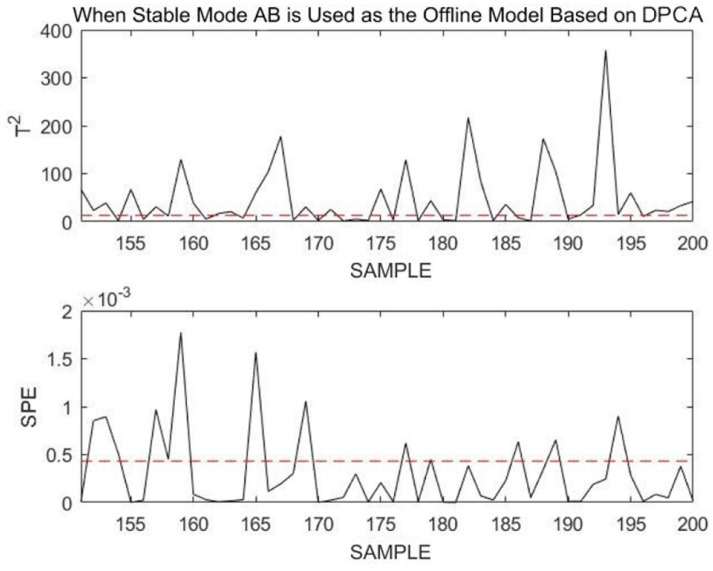
Monitoring 151st to 180th sample points using transition mode AB as an offline model Base on DPCA.

After modeling with DPCA and monitoring the 151st to 180th sample points of the online dataset, the obtained results are shown in Figure 20.

**Figure 21:**
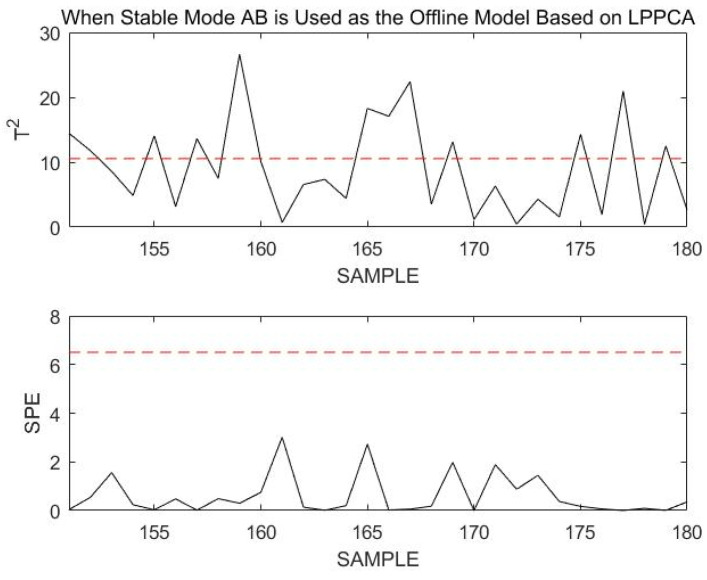
Monitoring 151st to 180th sample points using transition mode AB as an offline model Base on LPPCA.

After modeling using LPPCA and monitoring the 151st to 180th sample points of the online dataset, the obtained results are shown in Figure 21.

Using DLPPCA, DPCA, and LPPCA, the fault detection rate (FDR) and missed alarm rate (MAR) obtained when monitoring this transitional mode failure are shown in the following Table 4.

When the transition mode fails, the missed alarm rate (MAR) for modeling and monitoring with DPCA or LPPCA are much higher than that with DLPPCA. By comparison, the superiority of the DLPPCA method is proven. Generally, the DLPPCA method presented in this paper performs better on dynamic transition mode data and is more suitable for modeling and monitoring multimodal processes. Compared with DPCA, the accuracy of DLPPCA for fault monitoring is higher. This is because the combined use of the LPP method enables the extraction of a manifold structure that is more representative of the essential characteristics of the data while maintaining the nonlinear structure. DLPPCA fully considers both the global Euclidean structure and the local neighborhood structure of the dataset, instead of considering only one of these aspects. The false alarm rate of LPPCA for fault monitoring is much higher than that of DPCA and DLPPCA. This is due to the dynamic characteristics of autocorrelation and cross-correlation of process variables in real industrial processes. The traditional PCA-based approach is unable to extract dynamic relationships from the data, which makes it difficult to reveal the types of relationships between measured variables.

Next, another comparative simulation is used to illustrate the necessity of modeling stable modes and transition modes separately. This comparative simulation uses the DLPPCA method to perform overall offline modeling on the process containing stable mode A, stable mode B, transition mode AB, and transition mode BA without distinguishing between them.

**Figure 22:**
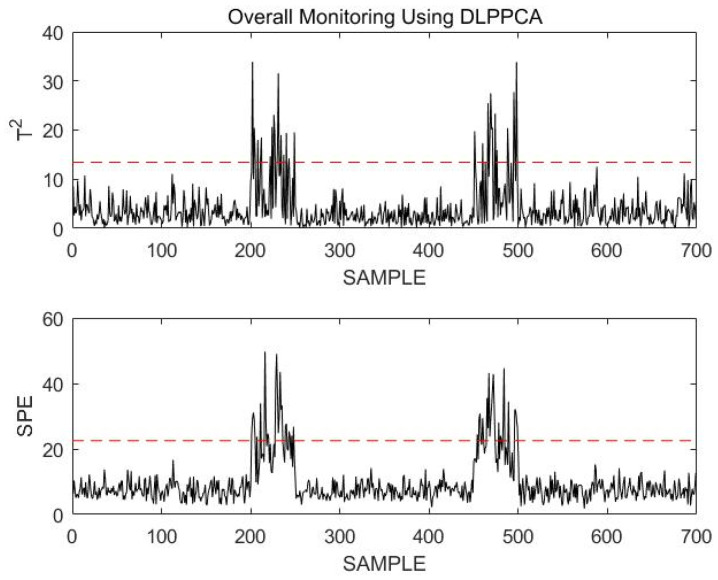
Overall monitoring using DLPPCA.

On this basis, online data with the same trend as that of the corresponding offline data are selected for monitoring; there are 700 sample points in the online dataset. At the 200th sample point, stable mode A changes to transition mode AB, and after 50 additional sample points, stable mode B is entered. At the 450th sample point, stable mode B switches to transition mode BA and then becomes stable mode A after 50 more sample points. All online data are monitored, and the results are shown in Figure 22.

It was clearly seen that the normal transition mode process was incorrectly identified as a fault during the overall monitoring process. This is not the case when the stable modes and transition modes are modeled and monitored separately. The necessity of modeling stable modes and transition modes separately can be demonstrated.

Through the above simulation using an actual dataset from a power plant motor, the following can be proven:

1. The DLPPCA method is more accurate than existing methods when modeling and monitoring transition modes. Compared with DPCA and LPPCA, the DLPPCA pro-posed in this paper has higher modeling accuracy. For transitional mode faults that are difficult to accurately monitor with other methods, accurate results can also be obtained by using DLPPCA.
2. Modeling stable modes and transition modes separately can improve the accuracy of online monitoring. If the multimodal process is indiscriminately modeled as a whole, the normal transitional modal process can easily be mismonitored as a fault by an online monitoring approach. Modeling stable modes and transition modes separately enables us to avoid such errors and make online monitoring more accurate.

## 6. Conclusions

In this paper, a new multimodal process detection method is presented. In the offline phase, the VLSW-MADF test is used to identify the inherent modes, separating the stable modes from the transition modes. Then, based on the results of mode identification, the stable modes and the transition modes are modeled separately using the proposed DLPPCA method, and the confidence limit and matching matrix of each mode are saved for online mode recognition and monitoring. The VLSW-MADF test is fast and accurate in mode identification, and DLPPCA performs better on transitional mode data than traditional methods. In the online monitoring phase, this paper takes full advantage of the previous moment’s historical mode and presents a new online mode identification method; this method is discussed separately and online mode identification is performed only when necessary, which reduces the computational load and improves the efficiency of mode identification. The feasibility and efficiency of the proposed method have been evaluated through case studies involving the TE process and power plant data. Several comparisons and simulations have been made. The results show that the proposed multimodal process fault monitoring method based on the VLSW-MADF test and DLPPCA improve the efficiency and accuracy of multimodal data monitoring.

In previous research work, multiple PCA and multiple PLS are considered as the most classical methods for multimode process monitoring. Zhao [25,26] used historical data to build a single PCA or PLS model for each mode. However, this approach splits the useful information hidden between data sequences and is highly dependent on similarity measure algorithms. The proposed DLPPCA model in this paper considers the serial correlation of process data and makes full use of the global Euclidean structure and local neighborhood structure of the dataset by introducing manifold learning. The simulation results show that DLPPCA performs better than the conventional method on transition mode data. Meanwhile, the DLPPCA monitoring model can detect faults in time not only in steady mode but also in transition mode. For the problem of an unknown modeling data mode, Tan [29] uses variable-length sliding windows to extract the correlation changes of offline normal operation data and achieves the division of stable modal data and transitional modal data according to the similarity of correlation between windows. However, in the process of mode identification, the influence of the selection of the boundary parameter α on the mode identification accuracy and monitoring effect is enormous. The boundary parameter α needs to be selected by a large number of repeated experiments and expert experience. The VLSW-MADF test method proposed in this study innovatively uses the smoothness of the data as the basis for the identification of stable and transitional modes, and can accurately determine the onset of transitional modes.

Although the method in this paper achieves better results on two numerical simulation cases, there is still much room for improvement and some limitations. For example, we still perform dynamic feature extraction and analysis by constructing an augmented matrix with time lag properties. However, this method will increase the dimensionality of the data matrix and increase the computational effort. In addition, continuous learning or lifelong learning has become a key research focus in machine learning, and many researchers have introduced continuous learning into the field of process monitoring and fault diagnosis. For example, Zhang [48] investigated a single model with continuous learning capability to monitor continuous modes and achieved good results. In future research, the authors will consider improvements to existing algorithms and prefer to extend PCA to a framework of continuous learning or adaptive updating of the overall model to propose a more effective approach for industrial process monitoring.

## Figures and Tables

**Table 1:** Eight process continuous variables in TE process.

Serial Number	Variable Name
1	D Feed
2	E Feed
3	Product Sep Temp
4	Product Sep Underflow
5	Stripper Underflow
6	Reactor Coolant Temp
7	Component D in Reactor
8	Component H in Purge

**Table 2:** TE simulation case online monitoring results.

Statistics	FDR	FAR	MAR	Detection Delay
$T^{2}$	91%	0%	9%	5
*SPE*	87%	0%	13%	9

**Table 3:** Seven variables included in the steam feed pump system.

Serial Number	Variable Name
1	Small engine speed
2	The flow of the steam feed pump
3	The pressure of the feedwater header
4	Intake pressure of steam feed pump
5	Feedwater flow
6	The temperature of the feedwater header
7	High-pressure inlet feedwater pressure

**Table 4:** FDR and MAR for transitional mode failure monitoring using DLPPCA, DPCA, and LPPCA.

Methods	FDR		MAR	
	$T^{2}$	SPE	$T^{2}$	SPE
DLPPCA	76%	54%	24%	46%
DPCA	62%	26%	38%	74%
LPPCA	40%	0%	60%	100%

## Data Availability

The publicly available TE process dataset was analyzed in this study. These data can be found here. [http://web.mit.edu/braatzgroup/links.html (accessed on 10 March 2020)]. The power plant data presented in this study are available upon request from the corresponding author. The data are not publicly available due to intellectual property protection.

## Nomenclature

** A **	Projection matrix for manifold feature extraction	$T_{ti}$	Matching matrix for historical transition modes
D	A n×n diagonal matrix	$\tilde{T_{ti}}$	The first ω column vectors of $T_{ti}$
$D_{d}$	Distance vector between $T_{online\;}$ and $\tilde{T_{ti}}$	$T_{\alpha }{}^{2}$	The confidence limit for $T^{2}$
$D_{ii}$	Importance of each sample	$t_{s}$	The t-statistic of the ADF test
F	Low dimensional manifold = $\left[f_{1},f_{2},\ldots ,f_{n}\right]$	W	Weight matrix
H	Window length for the first stage	$W_{ij}$	Relationship between two samples
$H^{*}$	Smoothness matrix of the first stage	X	Sample set = ${\left[x_{m}\left(1\right),x_{m}\left(2\right),\ldots ,x_{m}\left(n\right)\right]}^{T}$
I	Unit matrix	$X^{*}$	Sample set after dynamic expansion
k	Minimum number of generalized eigenvalues	$\bar{X}$	Trend variable
L	Window length for the second stage	$\tilde{X}$	Time series = ${\left[x_{1},x_{2},\ldots ,x_{n}\right]}^{T}$
$L_{P}$	A Laplacian matrix defined = D−W	${\bar{X}}_{new}$	Sample dataset to be reidentified and tested
l	Number of time lags	$x^{T}\left(t\right)$	The observation vector at moment t
m	Number of variables	∑	The covariance matrix of F
$m_{i}$	The matching value between $T_{online}$ and $\tilde{T_{ti}}$	β	Coefficient of time trend
n	Number of samples	$\gamma_{i}$	A trending term
P	The projection matrix when using PCA	δ	Coefficient of presenting process roots
ℝ	Set of real numbers	$\hat{\delta }$	The estimated value of δ
$SPE_{\alpha }$	The confidence limit for the SPE	${\hat{\sigma }}_{\delta }$	Standard errors
T	The feature data extracted by DLPPCA	$\varepsilon_{t}$	White noise sequence
$T_{online}$	The online matching matrix	η	An intercept constant called drift

## References

[1] Yin S., Li X., Gao H., Kaynak O. (2015). Data-Based Techniques Focused on Modern Industry: An Overview. IEEE Trans. Ind. Electron..

[2] Ji H., He X., Shang J., Zhou D. (2017). Incipient Fault Detection with Smoothing Techniques in Statistical Process Monitoring. Control. Eng. Pract..

[3] Peng X., Tang Y., Du W., Qian F. (2017). Multimode Process Monitoring and Fault Detection: A Sparse Modeling and Dictionary Learning Method. IEEE Trans. Ind. Electron..

[4] Quiñones-Grueiro M., Prieto-Moreno A., Verde C., Llanes-Santiago O. (2019). Data-Driven Monitoring of Multimode Continuous Processes: A Review. Chemom. Intell. Lab. Syst..

[5] Aldrich C., Auret L. (2013). Unsupervised Process Monitoring and Fault Diagnosis with Machine Learning Methods.

[6] Fan J., Wang W., Zhang H. AutoEncoder Based High-Dimensional Data Fault Detection System. Proceedings of the 2017 IEEE 15th International Conference on Industrial Informatics (INDIN).

[7] Zhao C., Gao F. (2014). Fault-Relevant Principal Component Analysis (FPCA) Method for Multivariate Statistical Modeling and Process Monitoring. Chemom. Intell. Lab. Syst..

[8] Zhang S., Zhao C. (2019). Hybrid Independent Component Analysis (H-ICA) with Simultaneous Analysis of High-Order and Second-Order Statistics for Industrial Process Monitoring. Chemom. Intell. Lab. Syst..

[9] Wang J., Zhong B., Zhou J.L. (2017). Quality-Relevant Fault Monitoring Based on Locality-Preserving Partial Least-Squares Statistical Models. Ind. Eng. Chem. Res..

[10] Lee J.-M., Yoo C., Choi S.W., Vanrolleghem P.A., Lee I.-B. (2004). Nonlinear Process Monitoring Using Kernel Principal Component Analysis. Chem. Eng. Sci..

[11] Ku W., Storer R.H., Georgakis C. (1995). Disturbance Detection and Isolation by Dynamic Principal Component Analysis. Chemom. Intell. Lab. Syst..

[12] Bakshi B.R. (1998). Multiscale PCA with Application to Multivariate Statistical Process Monitoring. AIChE J..

[13] Harrou F., Kadri F., Khadraoui S., Sun Y. (2016). Ozone Measurements Monitoring Using Data-Based Approach. Process Saf. Environ. Prot..

[14] Roweis S.T., Saul L.K. (2000). Nonlinear Dimensionality Reduction by Locally Linear Embedding. Science.

[15] Balasubramanian M., Schwartz E.L. (2002). The Isomap Algorithm and Topological Stability. Science.

[16] Belkin M., Niyogi P. (2003). Laplacian Eigenmaps for Dimensionality Reduction and Data Representation. Neural Comput..

[17] Wong W.K., Zhao H.T. (2012). Supervised Optimal Locality Preserving Projection. Pattern Recognit..

[18] Yu J. (2012). Local and Global Principal Component Analysis for Process Monitoring. J. Process Control.

[19] Luo L. (2014). Process Monitoring with Global–Local Preserving Projections. Ind. Eng. Chem. Res..

[20] Wu Y., Fu Z., Fei J. (2020). Fault Diagnosis for Industrial Robots Based on a Combined Approach of Manifold Learning, Treelet Transform and Naive Bayes. Rev. Sci. Instrum..

[21] Hwang D.-H., Han C. (1999). Real-Time Monitoring for a Process with Multiple Operating Modes. Control. Eng. Pract..

[22] Lane S., Martin E.B., Kooijmans R., Morris A.J. (2001). Performance Monitoring of a Multi-Product Semi-Batch Process. J. Process Control.

[23] Ma H., Hu Y., Shi H. (2012). A Novel Local Neighborhood Standardization Strategy and Its Application in Fault Detection of Multimode Processes. Chemom. Intell. Lab. Syst..

[24] Ng Y.S., Srinivasan R. (2009). An Adjoined Multi-Model Approach for Monitoring Batch and Transient Operations. Comput. Chem. Eng..

[25] Zhao S.J., Zhang J., Xu Y.M. (2004). Monitoring of Processes with Multiple Operating Modes through Multiple Principle Component Analysis Models. Ind. Eng. Chem. Res..

[26] Zhao S.J., Zhang J., Xu Y.M. (2006). Performance Monitoring of Processes with Multiple Operating Modes through Multiple PLS Models. J. Process Control.

[27] Kosanovich K.A., Piovoso M.J., Dahl K.S., MacGregor J.F., Nomikos P. Multi-Way PCA Applied to an Industrial Batch Process. Proceedings of the 1994 American Control Conference—ACC ’94.

[28] Kosanovich K.A., Dahl K.S., Piovoso M.J. (1996). Improved Process Understanding Using Multiway Principal Component Analysis. Ind. Eng. Chem. Res..

[29] Tan S., Wang F., Peng J., Chang Y., Wang S. (2012). Multimode Process Monitoring Based on Mode Identification. Ind. Eng. Chem. Res..

[30] Marx B.D. (1992). A User’s Guide to Principal Components. J. Am. Stat. Assoc..

[31] Xu D., Wang Y. (2007). An Automated Feature Extraction and Emboli Detection System Based on the PCA and Fuzzy Sets. Comput. Biol. Med..

[32] He K., Li X. (2019). Time–Frequency Feature Extraction of Acoustic Emission Signals in Aluminum Alloy MIG Welding Process Based on SST and PCA. IEEE Access.

[33] Zhang Y., Wang Z., Zhang J., Ma J. (2010). PCA Fault Feature Extraction in Complex Electric Power Systems. AECE.

[34] Yong-dong W., Dong-wei X., Peng P., Yi L., Gui-jun Z., Xiao X. (2019). Kernel PCA for Road Traffic Data Non-linear Feature Extraction. IET Intell. Transp. Syst..

[35] Li K., Wu Y., Song S., Sun Y., Wang J., Li Y. (2017). A Novel Method for Spacecraft Electrical Fault Detection Based on FCM Clustering and WPSVM Classification with PCA Feature Extraction. Proc. Inst. Mech. Eng. Part G J. Aerosp. Eng..

[36] He Q., Ding X., Pan Y. (2014). Machine Fault Classification Based on Local Discriminant Bases and Locality Preserving Projections. Math. Probl. Eng..

[37] Lv Y., Yuan R., Shi W. (2018). Fault Diagnosis of Rotating Machinery Based on the Multiscale Local Projection Method and Diagonal Slice Spectrum. Appl. Sci..

[38] Luo H., Tang Y.Y., Li C., Yang L. (2015). Local and Global Geometric Structure Preserving and Application to Hyperspectral Image Classification. Math. Probl. Eng..

[39] Zhang Z., Zhu X., Zhao J., Xu H. Image Retrieval Based on PCA-LPP. Proceedings of the 2011 10th International Symposium on Distributed Computing and Applications to Business, Engineering and Science.

[40] Zhang E.-H., Ma H.-B., Lu J.-W., Chen Y.-J. Gait Recognition Using Dynamic Gait Energy and PCA+LPP Method. Proceedings of the 2009 International Conference on Machine Learning and Cybernetics.

[41] Yang Q., Ba C., Li C., Wu D. An Ensemble Fault Diagnosis Approach for Multimodal Process. Proceedings of the 2017 IEEE International Conference on Signal Processing, Communications and Computing (ICSPCC).

[42] Richard P. (2009). Modified Fast Double Sieve Bootstraps for ADF Tests. Comput. Stat. Data Anal..

[43] Worden K., Iakovidis I., Cross E.J. (2021). New Results for the ADF Statistic in Nonstationary Signal Analysis with a View towards Structural Health Monitoring. Mech. Syst. Signal Process..

[44] Aylar E., Smeekes S., Westerlund J. (2019). Lag Truncation and the Local Asymptotic Distribution of the ADF Test for a Unit Root. Stat. Pap..

[45] Vosloo J., Uren K.R., van Schoor G., Auret L., Marais H. (2020). Exergy-Based Fault Detection on the Tennessee Eastman Process. IFAC-Pap. Line.

[46] Chen D., Li Z., He Z. Research on Fault Detection of Tennessee Eastman Process Based on PCA. Proceedings of the 2013 25th Chinese Control and Decision Conference (CCDC).

[47] Li H., Xiao D. (2011). Fault Diagnosis of Tennessee Eastman Process Using Signal Geometry Matching Technique. EURASIP J. Adv. Signal Process..

[48] Zhang J., Zhou D., Chen M. (2021). Monitoring Multimode Processes: A Modified PCA Algorithm with Continual Learning Ability. J. Process Control.

